# Intra-arterial thrombolysis agents following endovascular thrombectomy for acute ischemic stroke: a network meta-analysis

**DOI:** 10.3389/fphar.2026.1820597

**Published:** 2026-07-01

**Authors:** Jing Huang, Le-Le Qin, Pan Tao, Chi Meng, Wei Chen, Wen-Ye Wu, Yu-Qin Ran, Chang-Qing Zhou

**Affiliations:** 1 Department of Neurology, Bishan Hospital of Chongqing Medical University, Chongqing, China; 2 Department of Nephrology, Guangdong Provincial People’s Hospital (Guangdong Academy of Medical Sciences), Guangzhou, China

**Keywords:** endovascular treatment, intra-arterial thrombolysis, ischemic stroke, large-vessel occlusion, network meta-analysis

## Abstract

**Objective:**

Endovascular thrombectomy (EVT) is the standard treatment for acute ischemic stroke caused by large-vessel occlusion (AIS-LVO), but incomplete microvascular reperfusion often limits recovery. The 2026 American Heart Association/American Stroke Association guideline suggests that adjunctive intra-arterial thrombolysis (IAT) may be considered after successful EVT to target residual microthrombi, yet the comparative efficacy and safety of different IAT strategies remain unclear. This study therefore compared the efficacy and safety of various IAT strategies following successful EVT in AIS-LVO.

**Methods:**

We systematically searched MEDLINE, Embase, and Cochrane CENTRAL from their inception through 26 February 2026, for randomized controlled trials (RCTs) comparing EVT alone or with IAT. A Bayesian network meta-analysis using a fixed-effects model was conducted.

**Results:**

Seven RCTs including 2,131 patients were analyzed. Compared with EVT alone, EVT + 0.225 mg/kg alteplase (ALT) or 0.125 mg/kg tenecteplase (TNK) significantly improved excellent functional outcome (modified Rankin Scale [mRS] 0–1) (OR 1.95, 95% CrI 1.32–2.89; OR 1.91, 95% CrI 1.12–3.27), while EVT + 0.0625 mg/kg TNK was associated with a significantly higher risk of any intracranial hemorrhage (aICH) (OR 1.53, 95% CrI 1.13–2.07). No statistically significant differences were observed for functional independence (mRS 0–2), favorable outcome (mRS 0–3), symptomatic intracranial hemorrhage (sICH), or 90 day mortality. Surface under the cumulative ranking curve (SUCRA) analysis indicated that EVT + 0.225 mg/kg ALT ranked highest for functional independence (mRS 0–2), but also ranked higher for the risk of 90 day mortality. In contrast, EVT + 0.0625 mg/kg TNK ranked highest for favorable outcome (mRS 0–3), while also ranking highest for the risk of sICH and aICH.

**Conclusion:**

Not all IAT strategies showed statistically significant efficacy, but EVT + 0.225 mg/kg ALT or 0.125 mg/kg TNK significantly improved excellent functional outcomes. SUCRAs suggested that EVT + 0.225 mg/kg ALT may provide broader functional benefits with a lower risk of sICH. Further confirmation in head-to-head RCTs is warranted.

**Systematic Review Registration:**

https://www.crd.york.ac.uk/PROSPERO/view/CRD420251250195, identifier CRD420251250195.

## 1 Introduction

Acute ischemic stroke (AIS) remains one of the leading causes of death and long-term disability worldwide, imposing a substantial global health and socioeconomic burden (Feigin et al., 2025; GBD 2021 Stroke Risk Factor Collaborators, 2024; eClinicalMedicine, 2023). Among all subtypes, large-vessel occlusion (LVO) stroke accounts for approximately one-third of ischemic strokes but contributes to the majority of stroke-related mortality and severe disability (de Havenon et al., 2024; Malhotra et al., 2017; Rocha et al., 2019). Endovascular thrombectomy (EVT) has revolutionized the treatment of AIS-LVO, achieving high rates of large-vessel recanalization and becoming the standard of care for eligible patients (Campbell et al., 2015; Goyal et al., 2016; Berkhemer et al., 2015). However, despite angiographic success, a considerable proportion of patients fail to attain functional independence at 90 days (Lin et al., 2019). Recent studies indicate that these differences may be partly explained by the no-reflow phenomenon, characterized by microvascular perfusion failure despite complete macrovascular recanalization (Sun et al., 2024; Ng et al., 2022). Experimental and imaging evidence suggest that distal embolization, microthrombi formation, endothelial injury, and pericyte-mediated capillary constriction contribute to this microcirculatory dysfunction, which remains incompletely addressed by current reperfusion strategies (Sun et al., 2024; Zhang et al., 2024; Shrouder et al., 2024).

According to the 2026 American Heart Association/American Stroke Association guideline for the early management of acute ischemic stroke (Prabhakaran et al., 2026), the use of salvage technical adjuncts, including intra-arterial thrombolysis (IAT), may be considered in patients who have achieved successful EVT (modified Thrombolysis in Cerebral Infarction [mTICI] ≥2b) to improve cerebral reperfusion and 90 day functional outcomes (Class IIb, LOE B-R). In this context, adjunctive IAT following EVT has emerged as a potential approach to dissolve residual distal emboli and improve microvascular reperfusion (Diprose et al., 2021; Nie et al., 2023). Several randomized controlled trials (RCTs) (Renú et al., 2022; Liu et al., 2025; Huang et al., 2025; Hu et al., 2025; Miao et al., 2025; Writing Committee for the PEARL Investigators et al., 2025; Hou et al., 2025), including CHOICE, POST-UK, POST-TNK, ATTENTION-IA, ANGEL-TNK, PEARL, and DATE, have explored this strategy, yielding mixed or inconclusive findings. Recent conventional meta-analyses pooling these RCTs suggested that IAT may modestly enhance functional outcomes without significantly increasing hemorrhagic risk (Jiang et al., 2025; Guo et al., 2025). However, some prior analyses incorporated conference abstracts or preliminary reports before full peer-reviewed publication. In addition, no study has yet comprehensively compared the relative efficacy and safety of different IAT agents or their optimal dosing regimens.

To guide agent selection and optimize post-EVT reperfusion strategies, we conducted a Bayesian network meta-analysis (NMA) to compare the efficacy and safety of intra-arterial alteplase (ALT), tenecteplase (TNK, at various doses), and urokinase (UK) following successful EVT in patients with AIS-LVO.

## 2 Methods

### 2.1 Protocol and registration

Our analysis was guided by principles of the PRISMA (Preferred Reporting Items for Systematic Reviews and Meta-Analyses) Extension Statement (Hutton et al., 2015). The study protocol has been registered in PROSPERO (registration number: CRD420251250195).

### 2.2 Search strategy and data extraction

A comprehensive literature search (strategy in Supplementary Appendix S1) was conducted across MEDLINE (via PubMed), Embase, and the Cochrane Central Register of Controlled Trials from inception to 26 February 2026, which was the date of the final search. Additionally, we manually screened the reference lists of relevant meta-analyses, systematic reviews, and conference abstracts to identify ongoing, unpublished, or additional eligible studies, and searched the websites of major stroke journals to capture recently published RCTs not yet indexed in bibliographic databases (e.g., the PEARL trial, JAMA Online First, 13 October 2025) (Writing Committee for the PEARL Investigators et al., 2025). Following duplicate removal, two independent reviewers (J.H. and L.-L.Q.) conducted the title/abstract screening and data extraction. The extracted data encompassed study characteristics such as authorship, design, publication year, country, sample size, interventions, and outcomes. Any disagreements were resolved through consensus or, when necessary, by arbitration from a third reviewer (C.-Q.Z.). Efforts were made to acquire missing data by contacting the original investigators.

### 2.3 Selection criteria

The search strategy was based on the PICOS framework (P: population/patient, I: intervention, C: comparison, O: outcome, S: study design) (Moher et al., 2009). Eligible studies included adult patients with AIS-LVO who achieved successful EVT, defined as an expanded Thrombolysis in Cerebral Infarction (eTICI) score ≥2b50, based on angiographic assessment of downstream tissue reperfusion (indicating ≥50% reperfusion of the affected territory). This threshold corresponds broadly to mTICI 2b–3 and is commonly used to define successful reperfusion in endovascular trials. The intervention of interest was adjunctive IAT administered immediately after EVT, and the comparator was EVT alone. Outcomes of interest were predefined and are described in detail in Section 2.4. Only RCTs were included as the eligible study design; studies not designed as RCTs were excluded.

### 2.4 Outcomes

The study assessed both efficacy and safety outcomes. The primary efficacy endpoint was the proportion of participants who achieved excellent outcome, defined as a modified Rankin Scale (mRS) score of 0–1 at 90 days. Secondary efficacy outcomes included the proportion of participants who achieved functional independence (mRS 0–2) and favorable outcome (mRS 0–3) at 90 days. Safety outcomes included all-cause mortality at 90 days, as well as the incidence of symptomatic intracranial hemorrhage (sICH) and any intracranial hemorrhage (aICH) within 48 h. sICH was defined according to the criteria reported in the original trials, including the modified Heidelberg classification and the ECASS III criteria or related variants. When multiple definitions of sICH were available within a study, the definition used for the main analysis in the original report was adopted. The assessment time windows for sICH/aICH also varied across studies (typically 24–48 h); therefore, we adopted the definitions and time windows as reported in each trial.

### 2.5 Risk of bias assessment

Risk of bias was independently assessed by two reviewers (J.H. and P.T.) who used the Cochrane Risk of Bias Tool for randomized clinical trials (RoB 2) (Sterne et al., 2019). The certainty of the evidence was assessed using the Confidence in Network Meta-Analysis framework (CINeMA; accessible at https://cinema.ispm.unibe.ch/), which evaluates confidence in NMA estimates across six key domains: within-study bias, reporting bias, indirectness, imprecision, heterogeneity, and incoherence (Nikolakopoulou et al., 2020; Papakonstantinou et al., 2020). Any discrepancies in the CINeMA assessments were resolved through consensus discussions among the reviewers.

### 2.6 Statistical analysis

The comparative effectiveness of the interventions was evaluated using a Bayesian NMA in line with recommended guidelines (Dias et al., 2013; van Valkenhoef et al., 2012). This methodology synthesizes both direct and indirect evidence, facilitating the coherent comparison of multiple interventions within a single framework. All Bayesian computations were carried out in the R statistical environment (v4.3.3) utilizing the gemtc package (Hu et al., 2020). The model was fitted using Markov chain Monte Carlo methods with a Gibbs sampler, involving four independent chains. Each chain underwent a burn-in phase of 20,000 iterations, followed by 50,000 production iterations. Heterogeneity for pairwise comparisons was described using I^2^ statistics. For the Bayesian network meta-analysis, model selection between fixed-effect and random-effects models was guided by the between-study variance (τ^2^) and model fit indices (DIC, residual deviance). The agreement between direct and indirect evidence was examined, where feasible, via the node-splitting approach. Funnel plots were generated in R to explore potential publication bias, and Egger’s test was additionally conducted to quantify small-study effects. Effect estimates for binary outcomes are expressed as odds ratios (ORs) accompanied by 95% credible intervals (CrIs). Intervention rankings were summarized using the surface under the cumulative ranking curve (SUCRA). For clarity, higher SUCRA values indicate greater efficacy for efficacy outcomes and higher probability of harm for safety outcomes, as models were explicitly coded for each outcome type.

Three sensitivity analyses were performed. First, we excluded the ATTENTION-IA trial (Hu et al., 2025), limiting the analysis to anterior-circulation occlusions. Second, we excluded the CHOICE trial (Renú et al., 2022), restricting the analysis to studies conducted exclusively in China. Third, we excluded studies (Renú et al., 2022; Miao et al., 2025; Writing Committee for the PEARL Investigators et al., 2025; Hou et al., 2025) in which not all patients achieved final eTICI ≥2c, thereby focusing on patients with near-complete (eTICI 2c) or complete (eTICI 3) reperfusion. Subgroup analyses examined whether prior intravenous thrombolysis (IVT) modified the effect of EVT + IAT, with trials classified at the trial level as pure (EVT + IAT without IVT) (Liu et al., 2025; Huang et al., 2025; Miao et al., 2025; Hou et al., 2025) or mixed (trials including patients with and without prior IVT) (Renú et al., 2022; Hu et al., 2025; Writing Committee for the PEARL Investigators et al., 2025). An additional subgroup analysis was conducted according to onset-to-puncture time (OTP), categorizing trials (Renú et al., 2022; Hu et al., 2025; Hou et al., 2025) with a median OTP ≤6 h and those (Liu et al., 2025; Huang et al., 2025; Miao et al., 2025) with a median OTP >6 h, to distinguish early versus delayed treatment timing.

## 3 Results

### 3.1 Baseline characteristics of the included literature

Our systematic search identified 3,957 records, of which seven RCTs (Renú et al., 2022; Liu et al., 2025; Huang et al., 2025; Hu et al., 2025; Miao et al., 2025; Writing Committee for the PEARL Investigators et al., 2025; Hou et al., 2025) met the eligibility criteria and were included in the NMA (Supplementary Figure S1). These trials collectively enrolled 2,131 patients with AIS-LVO and evaluated six interventions, consisting of EVT alone and five adjunctive IAT regimens: 0.225 mg/kg ALT, 100,000 IU UK, and three TNK doses (0.0313 mg/kg, 0.0625 mg/kg, and 0.125 mg/kg). Specifically, the CHOICE (Renú et al., 2022) and PEARL (Writing Committee for the PEARL Investigators et al., 2025) trials evaluated intra-arterial ALT at 0.225 mg/kg; the DATE (Hou et al., 2025) trial compared TNK doses of 0.0313 mg/kg and 0.0625 mg/kg; POST-TNK (Huang et al., 2025) and ATTENTION-IA (Hu et al., 2025) assessed TNK at 0.0625 mg/kg; ANGEL-TNK (Miao et al., 2025) investigated TNK at 0.125 mg/kg; and POST-UK (Liu et al., 2025) evaluated intra-arterial UK at 100,000 IU. Bridging IVT was permitted in ATTENTION-IA, CHOICE, and PEARL, but not in the DATE, POST-TNK, POST-UK, or ANGEL-TNK trials.

The included studies exhibited some variation in stroke location and geographic setting. ATTENTION-IA focused on posterior circulation stroke, CHOICE included both anterior and posterior circulation strokes, and the remaining five trials enrolled patients with anterior circulation stroke. Geographically, CHOICE was conducted in Spain, while all other trials were performed in China. Patient characteristics were generally comparable across trials. In most trials, the reported mean or median age was approximately 65–73 years, baseline stroke severity was generally comparable, with NIHSS scores typically between 14 and 17, and the proportion of male participants was approximately 46.2%–72.6%. Notably, ATTENTION-IA enrolled a cohort with a higher percentage of male participants and more severe neurological deficits, whereas the other trials demonstrated more balanced baseline distributions. Detailed characteristics of the included RCTs are provided in Table 1.

**TABLE 1:** Baseline characteristics of included studies in the network meta-analysis.

Trials (years)	Country	Design	Occlusion site	Study arm (n)	Median age, years (IQR)	Male %	Median NIHSS (IQR)	Median OTP, min (IQR)	Median OTR, min (IQR)	IVT %	ASPECTS (IQR)	Final eTICI after EVT, %		
												2b50/67	2c	3
CHOICE (Renú et al., 2022) (2022)	Spain	Double-blinded	ICA, M1 M2, PCA	EVT + 0.225 mg/kg ALT (61)	73 (71–76)	54.0	14 (8–20)	261 (125–630)	306 (208–672)	51.7	9 (9–10)	56.0	44.0^b^	
				EVT (52)	73 (69–77)	54.0	14 (10–20)	292 (190–596)	345 (237–609)	51.9	10 (9–10)	60.0	40.0^b^	
POST-UK (Liu et al., 2025) (2025)	China	Open-label, blinded endpoint	ICA, M1 M2	EVT + 100,000 IU UK (267)	69 (59–77)	60.7	15 (11–19)	462 (260–730)	523 (312–779)	0	8 (7–9)	0	36.0	62.9
				EVT (267)	68 (58–76)	55.8	15 (10–19)	465 (255–753)	524 (318–817)	0	8 (7–9)	0	33.7	65.5
POST-TNK (Huang et al., 2025) (2025)	China	Open-label, blinded endpoint	ICA, M1 M2	EVT + 0.0625 mg/kg TNK (269)	69 (59–76)	57.2	15 (11–20)	413 (249–694)	500 (305–754)	0	8 (7–9)	0	37.5	62.1
				EVT (271)	69 (59–76)	60.9	15 (10–20)	415 (251–740)	490 (324–809)	0	8 (7–9)	0	37.6	61.6
ATTENTION-IA (Hu et al., 2025) (2025)	China	Open-label, blinded endpoint	BA, VA P1	EVT + 0.0625 mg/kg TNK (104)	65 ± 11^a^	80.8	20 (12–35)	276 (186–420)	/	26.9	9 (8–10)	0	11.5	69.2
				EVT (104)	67 ± 11^a^	73.0	23 (14–35)	354 (174–468)	/	24.0	8 (8–10)	0	15.4	75.0
ANGEL-TNK (Miao et al., 2025) (2025)	China	Open-label, blinded endpoint	ICA, M1 M2	EVT + 0.125 mg/kg TNK (126)	72 (61–80)	54.0	15 (12–19)	470 (330–738)	576 (399–841)	0	7 (6–8)	69.1	22.2	8.7
				EVT (129)	72 (62–79)	56.6	16 (12–19)	498 (342–732)	651 (421–930)	0	7 (6–8)	59.7	29.5	9.3
PEARL (Writing Committee for the PEARL Investigators et al., 2025) (2025)	China	Open-label, blinded endpoint	ICA, M1 M2	EVT + 0.225 mg/kg ALT (164)	66 ± 13^a^	69.4	15 (11–18)	/	402 (256–673)	41.7	9 (7–10)	60.4	34.2	5.5
				EVT (160)	65 ± 13^a^	72.6	15 (11–17)	/	403 (239–675)	42.1	9 (7–10)	55.6	31.9	12.5
DATE (Hou et al., 2025) (2025)	China	Open-label, blinded endpoint	ICA, M1 M2	EVT + 0.0625 mg/kg TNK (46)	71 (61–77)	63.0	17 (12–20)	342 (185–623)	410 (259–748)	0	8 (6–9)	23.9	32.6	43.5
				EVT + 0.0313 mg/kg TNK (46)	71 (60–76)	65.2	17 (10–20)	330 (205–648)	404 (262–725)	0	9 (8–9)	26.1	39.1	34.8
				EVT (65)	71 (56–78)	46.2	17 (12–20)	322 (232–635)	384 (288–677)	0	8 (7–9)	26.2	32.3	41.5

Abbreviations:ALT, alteplase; BA, basilar artery; EVT, endovascular treatment; ICA, internal carotid artery; M1, main trunk of the middle cerebral artery; M2, first-order branch of the main trunk of the middle cerebral artery; OTP, Onset-to-Puncture time; OTR, Onset-to-Randomisation time; P1, first segment of the posterior cerebral artery; PCA, posterior cerebral artery; TNK, tenecteplase; UK, urokinase; VA, vertebral artery; TICI, thrombolysis in cerebral ischemia.

^a^ Mean value.

^b^ Final eTICI, after EVT (2c–3).

### 3.2 Quality of evidence and model assessment

**FIGURE 1:**
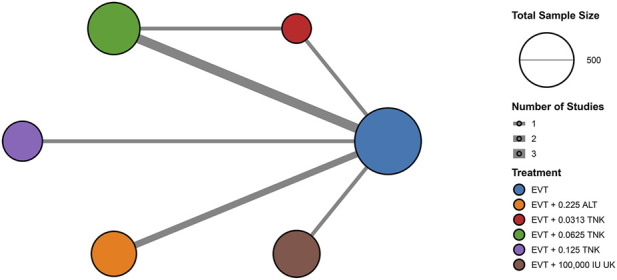
Network plot showing the total sample size for each treatment, with node size proportional to the number of participants receiving each treatment and line width proportional to the number of studies for each direct comparison. ALT, alteplase; EVT, endovascular treatment; TNK, tenecteplase; UK, urokinase.

To clearly illustrate the comparative relationships among interventions in the NMA, a network plot was generated (Figure 1). Model fit was evaluated for both random-effects (RE) and fixed-effect (FE) specifications, including deviance, effective number of parameters (pD), deviance information criterion (DIC), ratio, and I^2^ for each outcome (Supplementary Table S1). Heterogeneity was minimal across outcomes (I^2^ = 0–11%), with τ^2^ estimated as 0.211, 0.117, 0.127, 0.286, 0.689, and 0.246 for mRS 0–1, mRS 0–2, mRS 0–3, Mortality, sICH, and aICH, respectively. FE models consistently yielded slightly lower DIC values than RE models, while RE models added complexity (higher pD) without improving fit. Based on model comparisons, a fixed-effect specification was used for the primary analysis. Convergence was satisfactory, with potential scale reduction factors (PSRFs) consistently approaching 1, which was further supported by trace plots and posterior density plots (Supplementary Figure S2). According to the Cochrane Risk of Bias assessment (Supplementary Figures S3, S4), the CHOICE trial was judged as having “some concerns,” primarily due to issues related to the randomization process, whereas all other included studies were rated as low risk of bias.

According to the CINeMA framework, the certainty of evidence was rated as Moderate for 0.225 mg/kg ALT + EVT versus EVT alone and 0.0625 mg/kg TNK + EVT versus EVT alone for the primary efficacy outcome, and also Moderate for 0.0625 mg/kg TNK + EVT versus EVT alone for aICH. The certainty of evidence for all remaining comparisons was rated as Low. Summaries of the certainty assessments for outcomes are provided in Supplementary Table S2.

### 3.3 Efficacy outcomes

#### 3.3.1 Excellent outcome (mRS 0–1) at 90 days

**FIGURE 2:**
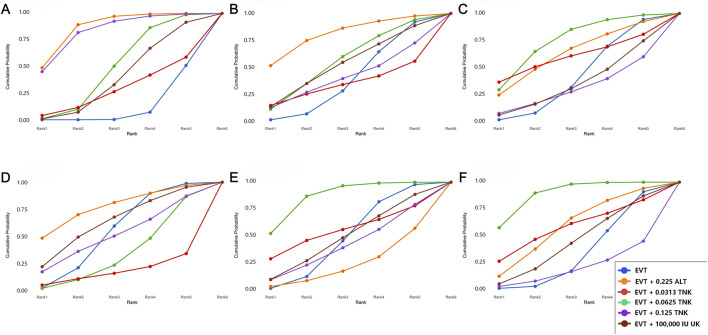
Surface under the cumulative ranking curve of available competing interventions for outcomes (higher SUCRA values indicate greater efficacy for efficacy outcomes and higher risk for safety outcomes). **(A)** = Excellent outcome (mRS 0–1) at 90 days, **(B)** = Functional independence (mRS 0–2) at 90 days, **(C)** = Favorable outcome (mRS 0–3) at 90 days, **(D)** = Mortality within 90 days, **(E)** = Symptomatic intracranial hemorrhage, **(F)** = Any intracranial hemorrhage. ALT, alteplase; EVT, endovascular treatment; mRS, modified Rankin Scale; SUCRA, Surface Under the Cumulative Ranking curve; TNK, tenecteplase; UK, urokinase.

For all efficacy outcomes, higher SUCRAs represent greater efficacy. Compared with EVT alone, EVT + 0.225 mg/kg ALT and EVT + 0.125 mg/kg TNK exhibited a statistically significant increase in efficacy for achieving an excellent outcome (OR = 1.95, 95% CrI = 1.32 to 2.89; OR = 1.91, 95% CrI = 1.12 to 3.27, respectively), while no significant differences were observed for EVT + 0.0313 mg/kg TNK, EVT + 0.0625 mg/kg TNK, or EVT + 100,000 IU UK. Moreover, all comparisons among the IAT agents revealed no statistically significant differences (Table 2). The SUCRA values indicated the following ranking for the potential best treatments: EVT + 0.225 mg/kg ALT (0.870), EVT + 0.125 mg/kg TNK (0.834), EVT + 0.0625 mg/kg TNK (0.493), EVT + 100,000 IU UK (0.400), EVT + 0.0313 mg/kg TNK (0.286), and EVT alone (0.117). All IAT agents demonstrated higher SUCRA rankings than EVT alone (Figure 2A).

**TABLE 2:** Efficacy outcomes of interventions represented by OR and 95% CrI in a fixed-effects model.

Intervention	EVT	EVT + ALT 0.225	EVT + TNK 0.0313	EVT + TNK 0.0625	EVT + TNK 0.125	EVT + UK 100,000 IU
Excellent outcome (mRS 0–1) at 90 days						
Rank	6	**1**	5	3	2	4
SUCRA	0.117	**0.870**	0.286	0.493	0.834	0.400
EVT + ALT 0.225	1.95 (1.32, 2.89)	​	​	​	​	​
EVT + TNK 0.0313	1.07 (0.51, 2.21)	0.55 (0.24, 1.26)	​	​	​	​
EVT + TNK 0.0625	1.31 (1.00, 1.73)	0.67 (0.42, 1.09)	1.22 (0.59, 2.59)	​	​	​
EVT + TNK 0.125	1.91 (1.12, 3.27)	0.98 (0.51, 1.90)	1.78 (0.73, 4.42)	1.45 (0.80, 2.66)	​	​
EVT + UK 100,000 IU	1.22 (0.87, 1.73)	0.63 (0.37, 1.06)	1.14 (0.51, 2.57)	0.93 (0.60, 1.45)	0.64 (0.34, 1.21)	​
Functional independence (mRS 0–2) at 90 days						
Rank	5	**1**	6	2	4	3
SUCRA	0.380	**0.805**	0.337	0.555	0.400	0.523
EVT + ALT 0.225	1.28 (0.88, 1.88)	​	​	​	​	​
EVT + TNK 0.0313	0.91 (0.45, 1.83)	0.71 (0.32, 1.57)	​	​	​	​
EVT + TNK 0.0625	1.08 (0.82, 1.42)	0.84 (0.53, 1.34)	1.19 (0.58, 2.42)	​	​	​
EVT + TNK 0.125	0.98 (0.60, 1.60)	0.77 (0.41, 1.43)	1.08 (0.46, 2.54)	0.91 (0.52, 1.60)	​	​
EVT + UK 100,000 IU	1.06 (0.75, 1.50)	0.83 (0.50, 1.39)	1.17 (0.54, 2.56)	0.99 (0.64, 1.53)	1.09 (0.60, 1.98)	​
Favorable outcome (mRS 0–3) at 90 days						
Rank	4	2	3	**1**	6	5
SUCRA	0.404	0.625	0.591	**0.741**	0.295	0.344
EVT + ALT 0.225	1.13 (0.76, 1.69)	​	​	​	​	​
EVT + TNK 0.0313	1.14 (0.54, 2.47)	1.01 (0.43, 2.41)	​	​	​	​
EVT + TNK 0.0625	1.20 (0.90, 1.60)	1.06 (0.65, 1.74)	1.05 (0.48, 2.24)	​	​	​
EVT + TNK 0.125	0.90 (0.55, 1.47)	0.80 (0.42, 1.50)	0.79 (0.32, 1.93)	0.75 (0.42, 1.33)	​	​
EVT + UK 100,000 IU	0.95 (0.66, 1.36)	0.84 (0.49, 1.44)	0.83 (0.35, 1.91)	0.79 (0.50, 1.26)	1.06 (0.57, 1.94)	​

Abbreviations:ALT, alteplase; EVT, endovascular treatment; mRS, modified Rankin Scale; TNK, tenecteplase; UK, urokinase.

Bold values indicate statistically significant odds ratios in pairwise comparisons or interventions ranked first in the surface under the cumulative ranking curve (higher SUCRA, values indicate greater efficacy).

#### 3.3.2 Functional independence (mRS 0–2) at 90 days

Compared with EVT alone, no IAT agents demonstrated statistically significant differences in functional independence, and no statistically significant differences were observed in the comparisons among the IAT agents. The SUCRAs indicate a ranking of the potential best treatments as EVT + 0.225 mg/kg ALT (0.805), EVT + 0.0625 mg/kg TNK (0.555), EVT + 100,000 IU UK (0.523), EVT + 0.125 mg/kg TNK (0.400), EVT alone (0.380), and EVT + 0.0313 mg/kg TNK (0.337) (Figure 2B).

#### 3.3.3 Favorable outcome (mRS 0–3) at 90 days

Compared with EVT alone, no IAT agents demonstrated statistically significant differences in favorable outcome, and no statistically significant differences were observed in the comparisons among the IAT agents. The SUCRAs indicate a ranking of the potential best treatments as EVT + 0.0625 mg/kg TNK (0.741), EVT + 0.225 mg/kg ALT (0.625), EVT + 0.0313 mg/kg TNK (0.591), EVT alone (0.404), EVT + 100,000 IU UK (0.344), and EVT + 0.125 mg/kg TNK (0.295) (Figure 2C).

### 3.4 Safety outcomes

#### 3.4.1 Mortality within 90 days

Importantly, for all safety outcomes, higher SUCRAs represent greater risk. For mortality outcome at 90 days, no statistically significant differences were observed among all intervention comparisons (Table 3). The SUCRAs indicate a ranking of mortality risk as EVT + 0.225 mg/kg ALT (0.774), EVT + 100,000 IU UK (0.637), EVT alone (0.546), EVT + 0.125 mg/kg TNK (0.516), EVT + 0.0625 mg/kg TNK (0.345), and EVT + 0.0313 mg/kg TNK (0.181) (Figure 2D).

**TABLE 3:** Safety outcomes of interventions represented by OR and 95% CrI in a fixed-effects model.

Intervention	EVT	EVT + ALT 0.225	EVT + TNK 0.0313	EVT + TNK 0.0625	EVT + TNK 0.125	EVT + UK 100,000 IU
Mortality within 90 days						
Rank	3	**1**	6	5	4	2
SUCRA	0.546	**0.774**	0.181	0.345	0.516	0.637
EVT + ALT 0.225	1.24 (0.71, 2.17)	​	​	​	​	​
EVT + TNK 0.0313	0.63 (0.23, 1.57)	0.51 (0.16, 1.48)	​	​	​	​
EVT + TNK 0.0625	0.88 (0.62, 1.23)	0.71 (0.37, 1.35)	1.38 (0.55, 3.84)	​	​	​
EVT + TNK 0.125	0.98 (0.54, 1.78)	0.79 (0.35, 1.81)	1.55 (0.52, 4.97)	1.12 (0.56, 2.22)	​	​
EVT + UK 100,000 IU	1.08 (0.69, 1.69)	0.87 (0.42, 1.78)	1.70 (0.62, 5.11)	1.23 (0.70, 2.15)	1.10 (0.52, 2.32)	​
Symptomatic intracranial hemorrhage						
Rank	4	6	2	**1**	5	3
SUCRA	0.473	0.226	0.544	**0.869**	0.408	0.480
EVT + ALT 0.225	0.65 (0.23, 1.74)	​	​	​	​	​
EVT + TNK 0.0313	1.16 (0.14, 6.23)	1.78 (0.18, 12.96)	​	​	​	​
EVT + TNK 0.0625	1.79 (0.97, 3.41)	2.78 (0.87, 9.52)	1.55 (0.29, 12.86)	​	​	​
EVT + TNK 0.125	0.89 (0.30, 2.59)	1.37 (0.31, 6.11)	0.78 (0.10, 7.99)	0.49 (0.14, 1.71)	​	​
EVT + UK 100,000 IU	1.00 (0.42, 2.39)	1.55 (0.42, 6.05)	0.87 (0.13, 8.34)	0.56 (0.19, 1.62)	1.13 (0.28, 4.60)	​
Any intracranial hemorrhage						
Rank	5	2	3	**1**	6	4
SUCRA	0.327	0.584	0.574	**0.890**	0.191	0.436
EVT + ALT 0.225	1.19 (0.79, 1.79)	​	​	​	​	​
EVT + TNK 0.0313	1.21 (0.54, 2.63)	1.02 (0.41, 2.45)	​	​	​	​
EVT + TNK 0.0625	1.53 (1.13, 2.07)	1.29 (0.78, 2.14)	1.26 (0.58, 2.84)	​	​	​
EVT + TNK 0.125	0.84 (0.48, 1.47)	0.71 (0.35, 1.42)	0.69 (0.27, 1.85)	0.55 (0.29, 1.04)	​	​
EVT + UK 100,000 IU	1.06 (0.72, 1.57)	0.90 (0.51, 1.58)	0.88 (0.37, 2.15)	0.69 (0.42, 1.14)	1.26 (0.64, 2.50)	​

Abbreviations:ALT, alteplase; EVT, endovascular treatment; TNK, tenecteplase; UK, urokinase.

Bold values indicate statistically significant odds ratios in pairwise comparisons or interventions ranked first in the surface under the cumulative ranking curve (higher SUCRA, values indicate higher risk).

#### 3.4.2 Symptomatic intracranial hemorrhage

For the outcome of sICH, no statistically significant differences were observed among all intervention comparisons. The SUCRA values indicate a ranking of the probability of having the highest bleeding risk as follows: EVT + 0.0625 mg/kg TNK (0.869), EVT + 0.0313 mg/kg TNK (0.544), EVT + 100,000 IU UK (0.480), EVT alone (0.473), EVT + 0.125 mg/kg TNK (0.408), and EVT + 0.225 mg/kg ALT (0.226) (Figure 2E).

#### 3.4.3 Any intracranial hemorrhage

Compared with EVT alone, only EVT + 0.0625 mg/kg TNK was associated with a statistically significant increase in the risk of aICH (OR = 1.53, 95% CrI = 1.13–2.07). No other IAT agent demonstrated a significant difference in risk. Furthermore, comparisons among the active agents revealed no statistically significant differences. The SUCRA values indicated the following ranking for the likelihood of having the highest bleeding risk: EVT + 0.0625 mg/kg TNK (0.890), EVT + 0.225 mg/kg ALT (0.584), EVT + 0.0313 mg/kg TNK (0.574), EVT + 100,000 IU UK (0.436), EVT alone (0.327), and EVT + 0.125 mg/kg TNK (0.191). Except for EVT + 0.125 mg/kg TNK, most IAT regimens had higher SUCRA values than EVT alone. This suggests a generally higher probability of bleeding risk with several adjunctive IAT strategies (Figure 2F).

### 3.5 Radar chart

**FIGURE 3:**
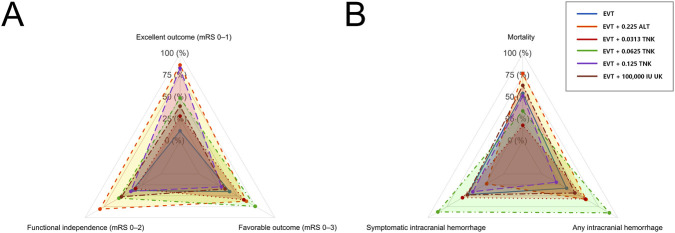
**(A)** Radar plot of efficacy outcomes including excellent functional outcome (mRS 0–1), functional independence (mRS 0–2), and favorable functional outcome (mRS 0–3). **(B)** Radar plot of safety outcomes including symptomatic intracranial hemorrhage (sICH), any intracranial hemorrhage (aICH), and mortality. In each panel, a larger coverage area indicates greater efficacy **(A)** or worse safety **(B)**. ALT, alteplase; EVT, endovascular treatment; mRS, modified Rankin Scale; SUCRA, Surface Under the Cumulative Ranking curve; TNK, tenecteplase; UK, urokinase.

The triangular radar charts (Figure 3) summarize the efficacy and safety profiles of the interventions. For efficacy, most IAT strategies showed radar profiles that fully encompassed EVT alone. EVT + 0.225 mg/kg ALT achieved the largest overall coverage, with notable advantages in excellent outcome (mRS 0–1) and functional independence (mRS 0–2). EVT + 0.125 mg/kg TNK and EVT + 0.0625 mg/kg TNK showed relative strengths in excellent (mRS 0–1) and favorable (mRS 0–3) outcomes, respectively, but with smaller overall coverage than EVT + 0.225 mg/kg ALT. For safety, EVT + 0.0625 mg/kg TNK had the largest radar chart coverage, driven by higher risks of sICH and aICH, followed by EVT + 0.225 mg/kg ALT, which was associated with increased mortality. EVT alone demonstrated a more favorable safety profile overall.

### 3.6 Two-dimensional scatterplot

**FIGURE 4:**
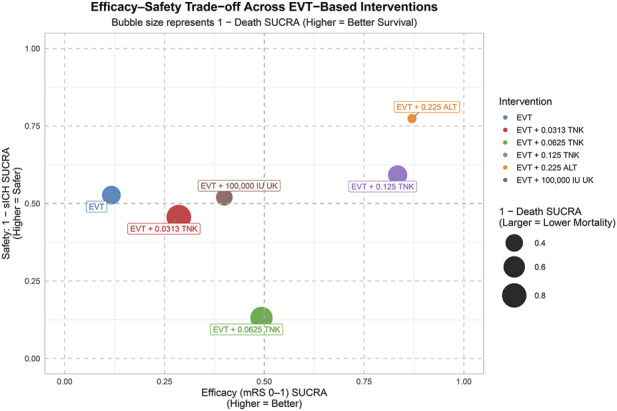
Two-dimensional scatter plot of the network comparing three outcomes. The x-axis represents the SUCRA value for excellent functional outcome (mRS 0–1), and the y-axis denotes 1 − SUCRA of sICH. Circle size corresponds to 1 − SUCRA of mortality. Interventions located in the upper-right quadrant with larger circles indicate more favorable overall profiles. ALT, alteplase; EVT, endovascular treatment; sICH, symptomatic intracranial hemorrhage; mRS, modified Rankin Scale; SUCRA, Surface Under the Cumulative Ranking curve; TNK, tenecteplase; UK, urokinase.

A two-dimensional scatter plot (Figure 4) integrating the SUCRA value for the primary efficacy outcome (mRS 0–1), 1 − SUCRA of sICH, and 1 − SUCRA of mortality was generated. EVT + 0.0313 mg/kg TNK was represented by the largest marker, indicating the greatest advantage in terms of mortality risk control. In contrast, EVT + 0.225 mg/kg ALT was positioned closest to the upper-right quadrant, suggesting the most advantageous overall balance between functional improvement and hemorrhagic risk control.

### 3.7 Assessment of heterogeneity, inconsistency, and publication bias

The assessment of heterogeneity for each outcome is shown in Supplementary Figure S5. Overall, heterogeneity was low across most outcomes (I^2^ < 25%), except for substantial heterogeneity observed in the comparisons of EVT + 0.225 mg/kg ALT versus EVT alone for 90 day mortality and sICH. The elevated I^2^ for mortality and sICH was primarily driven by discrepant results between the CHOICE (Renú et al., 2022) and PEARL (Writing Committee for the PEARL Investigators et al., 2025) trials, mainly attributable to the small sample size and zero-event occurrence in the CHOICE trial’s direct comparison, as well as differences in trial design. Sensitivity analysis excluding the CHOICE trial did not materially alter the results. Given that the EVT + 0.225 mg/kg ALT group exhibited low event rates for both outcomes and that neither trial demonstrated statistically significant differences compared with EVT alone, this heterogeneity is unlikely to materially affect the overall safety conclusions. Beyond heterogeneity, we also examined the structural consistency of the network. However, because the network contained no study-level closed loops, formal inconsistency assessment using the node-splitting approach was not feasible.

Publication bias and small-study effects were assessed using comparison-adjusted funnel plots. Both visual inspection and Egger’s test revealed no significant asymmetry (Supplementary Figure S6), suggesting no substantial publication bias. Nevertheless, given the limited statistical power of these methods when few studies are available, the results should be interpreted with appropriate caution.

### 3.8 Sensitivity analysis

After excluding the ATTENTION-IA (Hu et al., 2025) trial, which enrolled AIS-LVO patients with posterior circulation occlusions, no substantial changes were observed across any outcomes. A slight increase in the SUCRA of EVT + 0.0313 mg/kg TNK over EVT + 0.225 mg/kg ALT emerged for favorable outcome (mRS 0–3); however, the pairwise comparison remained statistically non-significant, with an OR close to 1, likely reflecting residual confounding inherent to the trial rather than a true treatment advantage. Furthermore, we performed an additional sensitivity analysis by excluding the CHOICE (Renú et al., 2022) trial, which recruited patients exclusively from Spain. This analysis was thereby restricted to trials conducted in China, and again, no statistically significant differences or shifts in SUCRA rankings were detected for any outcome. Finally, we excluded studies (Renú et al., 2022; Miao et al., 2025; Writing Committee for the PEARL Investigators et al., 2025; Hou et al., 2025) that included patients with post-EVT eTICI < 2c, restricting the analysis to those who achieved near-complete (eTICI 2c) or complete (eTICI 3) reperfusion. In this analysis, only functional independence (mRS 0–2) showed a higher SUCRA for EVT + 100,000 IU UK compared with EVT + 0.0625 mg/kg TNK, yet the corresponding comparison remained statistically non-significant with an OR close to 1, most likely attributable to reduced sample size rather than a true difference.

Taken together, these sensitivity analyses consistently demonstrated that the principal findings of our study were robust and remained largely unaffected by variations in study inclusion criteria.

### 3.9 Subgroup analysis

We conducted a subgroup analysis based on whether IVT was administered prior to EVT at the trial level. Four studies (Liu et al., 2025; Huang et al., 2025; Miao et al., 2025; Hou et al., 2025) that exclusively enrolled patients who did not receive IVT prior to EVT were classified as the pure subgroup (EVT + IAT). In contrast, three studies (Renú et al., 2022; Hu et al., 2025; Writing Committee for the PEARL Investigators et al., 2025) that included a mixture of patients with and without prior IVT were classified as the mixed subgroup, reflecting heterogeneity in pre-EVT treatment within those trials. This classification was intended to explore whether the presence of prior IVT at the trial level influenced the estimated treatment effects of adjunctive IAT. An additional subgroup analysis was performed based on median OTP, categorizing trials (Renú et al., 2022; Hu et al., 2025; Hou et al., 2025) with a median OTP ≤6 h and those (Liu et al., 2025; Huang et al., 2025; Miao et al., 2025) with a median OTP >6 h (OTP data were not available for the PEARL trial). This threshold was selected to distinguish early versus delayed treatment timing, which may influence the effectiveness and safety of reperfusion therapies. Because only a limited number of interventions overlapped between subgroups, the network structure was sparse; therefore, between-subgroup comparisons were summarized descriptively relative to the overall analysis.

In the pure EVT + IAT subgroup, five interventions were included (EVT alone, EVT + 0.0313 mg/kg TNK, EVT + 0.0625 mg/kg TNK, EVT + 0.125 mg/kg TNK, and EVT + 100,000 IU UK). Compared with the overall analysis, no statistically significant changes or SUCRA ranking shifts were observed across any outcomes. In the mixed subgroup, only three interventions were available (EVT alone, EVT + 0.225 mg/kg ALT, and EVT + 0.0625 mg/kg TNK). Notably, the comparison of EVT + 0.0625 mg/kg TNK versus EVT alone showed a reversal in the direction of effect as reflected by the odds ratios for functional independence (mRS 0–2), favorable outcome (mRS 0–3), and all-cause mortality (OR = 0.92, 95% CrI = 0.53 to 1.60; OR = 0.93, 95% CrI = 0.54 to 1.59; and OR = 1.05, 95% CrI = 0.57 to 1.94, respectively). Further stratified by OTP, in the OTP ≤6 h subgroup, four interventions were included (EVT alone, EVT + 0.225 mg/kg ALT, EVT + 0.0313 mg/kg TNK, and EVT + 0.0625 mg/kg TNK). For sICH, EVT + 0.225 mg/kg ALT was associated with a lower estimated risk compared with EVT alone, EVT + 0.0313 mg/kg TNK, and EVT + 0.0625 mg/kg TNK, with limited precision (OR estimates approached zero, suggesting zero events in some comparisons). In contrast, in the OTP >6 h subgroup (including EVT alone, EVT + 0.0625 mg/kg TNK, EVT + 0.125 mg/kg TNK, and EVT + 100,000 IU UK), no statistically significant differences or SUCRA ranking shifts were observed for any outcomes. All detailed subgroup analysis results are presented in Supplementary Table S3.

## 4 Discussion

This Bayesian NMA of RCTs suggests that some IAT strategies combined with EVT are associated with improved outcomes for the primary endpoint of excellent functional outcome (mRS 0–1), whereas not all IAT regimens demonstrated statistically significant benefit compared with EVT alone. Specifically, EVT + 0.225 mg/kg ALT and EVT + 0.125 mg/kg TNK demonstrated statistically significant superiority over EVT alone. For functional independence (mRS 0–2) and favorable outcome (mRS 0–3), both EVT + 0.225 mg/kg ALT and EVT + 0.0625 mg/kg TNK maintained directionally favorable effects relative to EVT alone, although the differences did not reach statistical significance. Regarding safety, no statistically significant differences were observed in the risks of 90 day mortality and sICH across the IAT strategies. However, EVT + 0.225 mg/kg ALT exhibited the highest mortality risk among all regimens, whereas EVT + 0.0625 mg/kg TNK significantly increased the risk of aICH and may also be associated with the highest risk of symptomatic intracranial hemorrhage.

In terms of functional outcomes, EVT + 0.225 mg/kg ALT and EVT + 0.125 mg/kg TNK were statistically significantly superior to EVT alone for achieving excellent functional outcome (mRS 0–1). EVT + 0.0625 mg/kg TNK reached borderline significance, likely reflecting increased statistical power from pooling three underpowered direct-comparison studies (Huang et al., 2025; Hu et al., 2025; Hou et al., 2025). No statistically significant differences were observed for functional independence (mRS 0–2) or favorable outcome (mRS 0–3) either between any IAT strategies and EVT alone or among different regimens. SUCRA rankings indicated that EVT + 0.225 mg/kg ALT performed best for mRS 0–1 and 0–2, while EVT + 0.0625 mg/kg TNK ranked highest for mRS 0–3. Overall, EVT + 0.225 mg/kg ALT showed the most favorable efficacy, followed by EVT + 0.125 mg/kg TNK, which ranked lowest for the more permissive favorable outcome. EVT + 0.0625 mg/kg TNK did not show a clear advantage over EVT alone for the primary endpoint. The more favorable ranking of ALT may be partly attributable to its continuous infusion, allowing sustained local drug exposure to residual microthrombi after EVT, whereas TNK is administered as a single bolus and may require higher doses to achieve comparable local concentrations (Meng et al., 2024; Huang et al., 2015; Rodriguez et al., 2024; Singh et al., 2024).

Moreover, subgroup analyses suggested that IVT may attenuate the potential functional benefit of EVT + 0.0625 mg/kg TNK and possibly increase mortality. However, such directional changes in non-significant estimates should be interpreted cautiously, as they may reflect random variation or residual confounding rather than true effect modification. Overall, the efficacy advantage of EVT + 0.0625 mg/kg TNK remains uncertain.

In terms of safety, the majority of IAT strategies did not demonstrate a statistically significant increase in risk. With respect to mortality, SUCRA rankings indicated that EVT + 0.225 mg/kg ALT was associated with the highest risk. Notably, this finding contrasts with the lower estimated risk of sICH observed with the same regimen, suggesting that mortality following EVT may be influenced by factors beyond hemorrhagic complications. Substantial heterogeneity was observed in the comparison between EVT + 0.225 mg/kg ALT and EVT alone, with the elevated I^2^ primarily driven by discrepant findings between the CHOICE (Renú et al., 2022) and PEARL (Writing Committee for the PEARL Investigators et al., 2025) trials. This inconsistency may be partly attributable to the small sample size of the CHOICE trial and the potential beneficial effects conferred by its double-blind, placebo-controlled design (Table 1). In addition, the relatively higher mortality observed in the EVT + 0.225 mg/kg ALT group in the PEARL trial may reflect its open-label design or, as described by the investigators, the possibility that “the increased procedural complexity associated with intra-arterial ALT administration may have contributed to higher mortality by prolonging procedure time and potentially compromising marginally perfused brain tissue.” Importantly, previous studies have reported that lower rates of symptomatic intracranial hemorrhage do not necessarily correspond to reduced mortality after EVT, suggesting a potential dissociation between hemorrhagic complications and overall clinical outcomes (Li et al., 2023; Chen et al., 2018). Consistent with these observations, mortality after EVT is widely recognized as a multifactorial outcome influenced by baseline stroke severity, onset-to-treatment time, infarct core size, collateral status, and comorbid conditions, which may not be fully balanced across studies. Therefore, the apparent discrepancy between hemorrhagic risk and mortality does not necessarily imply a direct causal relationship and should be interpreted with caution (Prabhakaran et al., 2026). This interpretation is further supported by the PEARL trial, in which higher baseline NIHSS scores and longer onset-to-randomization times may partly explain the higher mortality observed in the EVT + 0.225 mg/kg ALT group. Nevertheless, no study to date has clearly established a direct link between procedural complexity and increased mortality, and the SUCRA-based mortality ranking should be interpreted cautiously.

On the other hand, EVT + 0.0625 mg/kg TNK had the highest probabilities of sICH and aICH, and a statistically significant increase in aICH was observed compared with EVT alone. In contrast, EVT + 0.225 mg/kg ALT showed the lowest probability of sICH and demonstrated statistically significant differences compared with EVT alone or other IAT strategies in the OTP ≤6 h subgroup (although the observed ORs of 0.00 likely reflect the absence of events in the EVT + 0.225 mg/kg ALT group and the low overall event rate, rather than a true null risk). Previous evidence has suggested that ALT may increase the risk of sICH in extended treatment windows (Ahmad et al., 2025), indicating that the hemorrhagic risk of ALT may depend on treatment timing. Evidence from the NOR-TEST 2 Part A (Kvistad et al., 2022) trial similarly indicated that TNK thrombolysis was associated with higher risks of intracranial hemorrhage and mortality than ALT, leading to early termination of the trial. The elevated hemorrhagic risk with TNK may reflect its pharmacologic profile, including bolus administration, a longer half-life, and a greater propensity to disrupt the blood–brain barrier and induce vasogenic edema (Rodriguez et al., 2024; Singh et al., 2024; Kvistad et al., 2022). Notably, our NMA did not show a dose–response pattern for TNK. EVT + 0.125 mg/kg TNK had lower risks of sICH and aICH, likely reflecting study-level differences rather than a true pharmacologic effect. Only the ANGEL-TNK (Miao et al., 2025) trial contributed data for this dose, with a population that had lower overall hemorrhagic risk and more patients with lower post-EVT reperfusion grades (eTICI 2b50/67), which may have reduced reperfusion injury and subsequent hemorrhagic transformation (Li et al., 2016; Stebner et al., 2025; Mizuma et al., 2018). These factors likely explain the lower bleeding rates, so differences across TNK doses should not be interpreted as a protective effect, but as a consequence of limited and heterogeneous evidence.

To our knowledge, few studies have performed a Bayesian network meta-analysis based exclusively on randomized controlled trials to address this topic. Previous traditional meta-analyses were generally not designed to evaluate differences across specific IAT agents or dosing regimens, and therefore were limited in their ability to identify optimal drug selection and dosing strategies. These analyses typically relied on aggregated comparisons or simplified subgroup approaches, which may not adequately capture treatment-specific effects (Jiang et al., 2025; Guo et al., 2025; Palaiodimou et al., 2025). In addition, some of these studies incorporated unpublished data from RCTs such as ANGEL-TNK, PEARL, and DATE, which may have introduced additional uncertainty and limited the interpretability of the results. In contrast, the present study provides a more structured and comprehensive evaluation based on available randomized evidence, while incorporating the most recent data. According to the latest results from the CHOICE-2 phase III trial presented at the 2026 International Stroke Conference (Renú et al., 2026), the proportion of patients achieving excellent functional outcome (mRS 0–1) was 57.5% in the EVT + 0.225 mg/kg ALT group, compared with 42.5% in the EVT alone group (95% CI 5.7%–24.3%, p = 0.002). Mortality was slightly higher in the combined treatment group (12.1% vs. 6.4%), although the difference was not statistically significant. These findings are broadly consistent with the conclusions of our study.

As with any NMA, several limitations should be considered. First, the primary limitation relates to the insufficient number of RCTs. The entire evidence network was based on comparisons with EVT alone, and some comparisons (0.0313 mg/kg TNK vs. EVT alone; 0.125 mg/kg TNK vs. EVT alone; 100,000 IU UK vs. EVT alone) were informed by only a single RCT, which limits the assessment of consistency and the robustness of indirect comparisons. Second, there were variations in trial design and actual treatment practices across study centers (Table 1), including differences in blinding procedures, geographic origins and sex distribution of participants, post-recanalization eTICI scores, as well as the use of IVT and periprocedural concomitant medications. Notably, most included trials were conducted in Chinese populations, whereas CHOICE was conducted in Spain. Therefore, the generalizability of our findings to broader international populations should be interpreted with caution. Given the limited number of RCTs and the lack of individual patient-level data, we were also unable to assess whether sex-related, genetic, biochemical, or other population-specific factors modified treatment response. Furthermore, the definition of sICH differed among the included studies. Five studies (Liu et al., 2025; Huang et al., 2025; Hu et al., 2025; Miao et al., 2025; Writing Committee for the PEARL Investigators et al., 2025) applied the modified Heidelberg criteria, while others used either a more lenient variant or the ECASS III criteria. Reported time windows for sICH/aICH also varied (24–48 h); for this analysis, we used the definitions as reported in each trial. Finally, the CINeMA evaluation indicated low confidence in the evidence for most comparisons, mainly attributable to concerns in key domains such as imprecision and heterogeneity. Taken together, these limitations underscore the need for future large-scale, high-quality international RCTs to validate our findings and to further explore potential population-specific modifiers of treatment response.

## 5 Conclusion

In summary, this NMA compared IAT agents including ALT, different doses of TNK, and UK against EVT alone. Evidence remains limited, and these findings should be interpreted cautiously. Notably, not all IAT strategies demonstrated statistically significant efficacy, but EVT + 0.225 mg/kg ALT and EVT + 0.125 mg/kg TNK significantly improved excellent functional outcomes in patients with AIS-LVO compared with EVT alone. According to Bayesian ranking, EVT + 0.225 mg/kg ALT also performed well in functional independence and favorable outcome, while maintaining a favorable hemorrhagic safety profile, whereas EVT + 0.125 mg/kg TNK showed less consistent benefit across broader functional outcomes and an uncertain advantage in bleeding risk. Taken together, based on current RCT evidence, EVT + 0.225 mg/kg ALT may be considered a promising treatment option, and EVT + 0.125 mg/kg TNK a potential alternative. Further confirmation through rigorously designed head-to-head RCTs is warranted.

## Data Availability

The original contributions presented in the study are included in the article/Supplementary Material, further inquiries can be directed to the corresponding author.
