# Supplementary material for: Intra-arterial thrombolysis agents following endovascular thrombectomy for acute ischemic stroke: a network meta-analysis
Source: Front Pharmacol. 2026 Jul 1;17:1820597. doi: 10.3389/fphar.2026.1820597 (PMC13370159; doi:10.3389/fphar.2026.1820597)
Supplement: Supplementary file 2 [file DataSheet1.pdf]

Supplementary Material

Supplementary Appendix S1. Search Strategy used in our article.

Supplementary Figure S1. PRISMA flow chart.

Supplementary Figure S2. Trace plots and density plots.

Supplementary Figure S3. Cochrane risk of bias assessments for each included study.

Supplementary Figure S4. Risk of bias graph.

Supplementary Figure S5. Results of the heterogeneity analysis.

Supplementary Figure S6. Comparison-adjusted funnel plots for publication bias.

Supplementary Table S1. Model fit details including the random effect (RE) compared and the fixed effect (FE) model.

Supplementary Table S2. CINeMA Assessment.

Supplementary Table S3. Results of subgroup analyses.

Appendix S1. Search Strategy used in our article.

(a) <u>Search algorithm used in MEDLINE 505</u>

(("Intra-Arterial Thrombolysis"[tiab] OR "intraarterial thrombolysis"[tiab] OR "IAT"[tiab]) OR (Urokinase[tiab] OR Tenecteplase[tiab] OR TNK[tiab] OR Reteplase[tiab] OR "rt-PA"[tiab] OR alteplase[tiab] OR "recombinant tissue plasminogen activator"[tiab])) AND ("Thrombectomy"[MeSH] OR "Endovascular Procedures"[MeSH] OR thrombectomy[tiab] OR "mechanical thrombectomy"[tiab] OR EVT[tiab] OR "endovascular therapy"[tiab]) AND ("Stroke"[MeSH] OR "Cerebral Infarction"[MeSH] OR "acute ischemic stroke"[tiab] OR AIS[tiab] OR "large vessel occlusion"[tiab] OR LVO[tiab] OR "LVO stroke"[tiab] OR "AIS-LVO"[tiab]) AND (randomized[tiab] OR randomised[tiab] OR randomly[tiab] OR "randomized controlled trial"[pt] OR "controlled clinical trial"[pt] OR placebo[tiab] OR trial[tiab]) NOT (animals[mh] NOT humans[mh])

(b) <u>Search algorithm used in EMBASE 2918</u>

#1 'acute ischemic stroke'/exp OR 'acute ischemic stroke':ti,ab OR 'large vessel occlusion'/exp OR 'large vessel occlusion':ti,ab OR 'anterior circulation stroke'/exp OR 'anterior circulation stroke':ti,ab OR 'posterior circulation stroke'/exp OR 'posterior circulation stroke':ti,ab OR 'cerebral infarction'/exp OR 'cerebral infarction':ti,ab OR 'cerebral ischemia'/exp OR 'cerebral ischemia':ti,ab

#2 'urokinase'/exp OR 'urokinase':ti,ab OR 'tenecteplase'/exp OR 'tenecteplase':ti,ab OR 'tnk':ti,ab OR 'reteplase'/exp OR 'reteplase':ti,ab OR 'rt-pa':ti,ab OR 'recombinant tissue plasminogen activator'/exp OR 'recombinant tissue plasminogen activator':ti,ab OR 'intra-arterial':ti,ab OR 'mechanical thrombolysis'/exp OR 'mechanical thrombolysis':ti,ab OR 'thrombolytic therapy'/exp OR 'thrombolytic therapy':ti,ab

#3 'endovascular treatment'/exp OR 'endovascular treatment':ti,ab OR 'mechanical thrombectomy'/exp OR 'mechanical thrombectomy':ti,ab OR 'endovascular procedure'/exp OR 'endovascular procedure':ti,ab OR 'endovascular therapy':ti,ab OR 'endovascular thrombectomy':ti,ab OR 'intravascular procedure':ti,ab

#4 'randomized controlled trial'/exp OR 'randomized controlled trial':ti,ab OR

'randomised controlled trial':ti,ab OR 'randomized':ti,ab OR 'randomised':ti,ab OR 'randomly':ti,ab OR 'placebo':ti,ab OR 'trial':ti,ab

#5 #1 AND #2 AND #3 AND #4

(c) Search algorithm used in Cochrane Central Register of Controlled Trials 534

#1 (acute ischemic stroke):ti,ab,kw OR (large vessel occlusion):ti,ab,kw OR (anterior circulation stroke):ti,ab,kw OR (posterior circulation stroke):ti,ab,kw OR (cerebral infarction):ti,ab,kw

#2 (urokinase):ti,ab,kw OR (tenecteplase):ti,ab,kw OR (reteplase):ti,ab,kw OR ("rt-PA"):ti,ab,kw OR ("recombinant tissue plasminogen activator"):ti,ab,kw OR (intra-arterial):ti,ab,kw OR (mechanical thrombolysis):ti,ab,kw OR (thrombolytic therapy):ti,ab,kw

#3 (endovascular treatment):ti,ab,kw OR (mechanical thrombectomy):ti,ab,kw OR (endovascular procedure):ti,ab,kw OR (endovascular therapy):ti,ab,kw OR (endovascular recanalization):ti,ab,kw OR (endovascular thrombectomy):ti,ab,kw OR (intravascular procedure):ti,ab,kw

#4 ("randomized controlled trial"):ti,ab,kw OR ("randomised controlled trial"):ti,ab,kw OR (randomized):ti,ab,kw OR (randomised):ti,ab,kw OR (trial):ti,ab,kw OR (placebo):ti,ab,kw

#5 #1 AND #2 AND #3 AND #4

Figure S1. PRISMA flow chart.

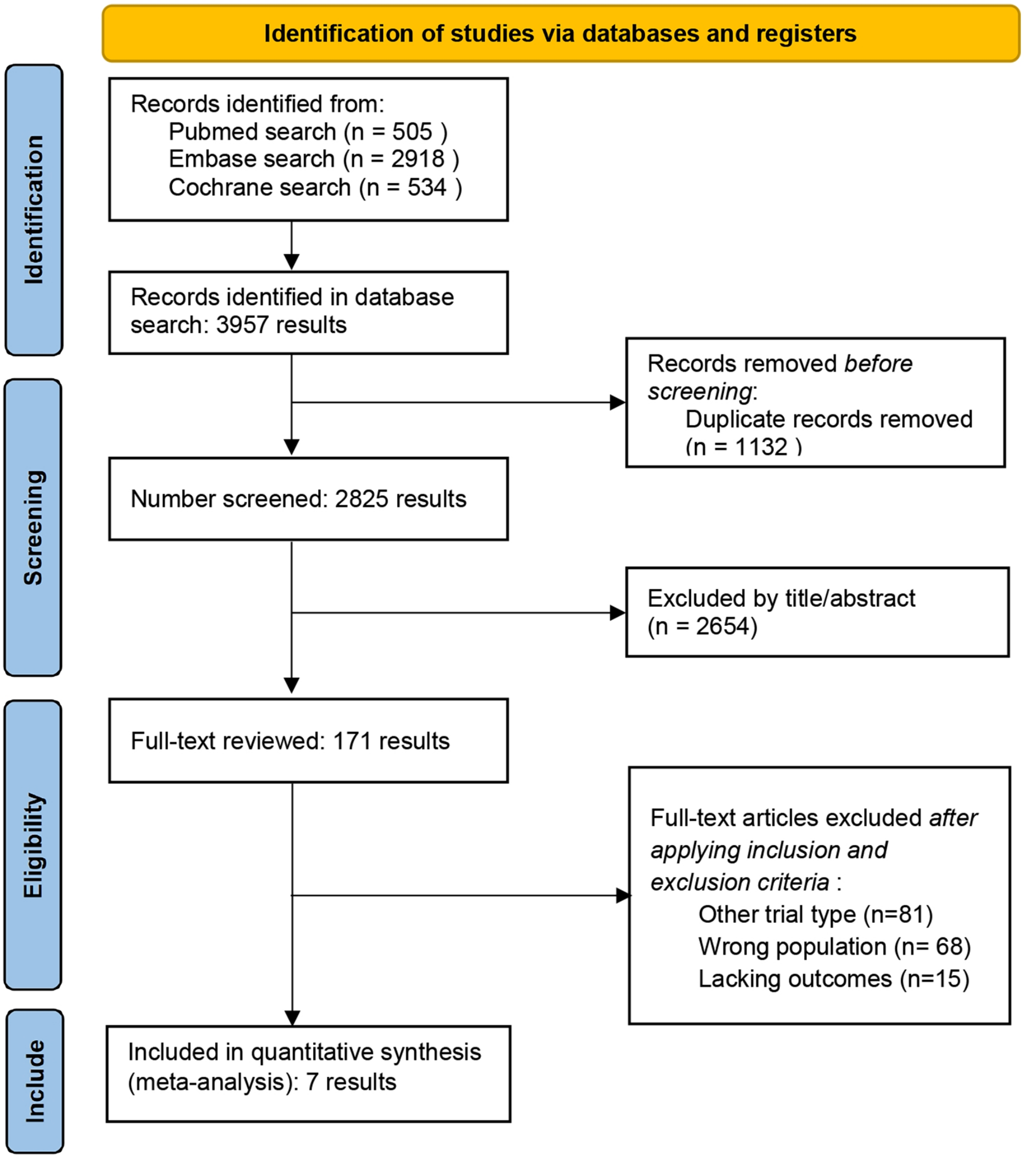

Figure S2. Trace plots and density plots.

(a) Trace plot and density plot for excellent outcome (mRS 0–1) at 90 days.

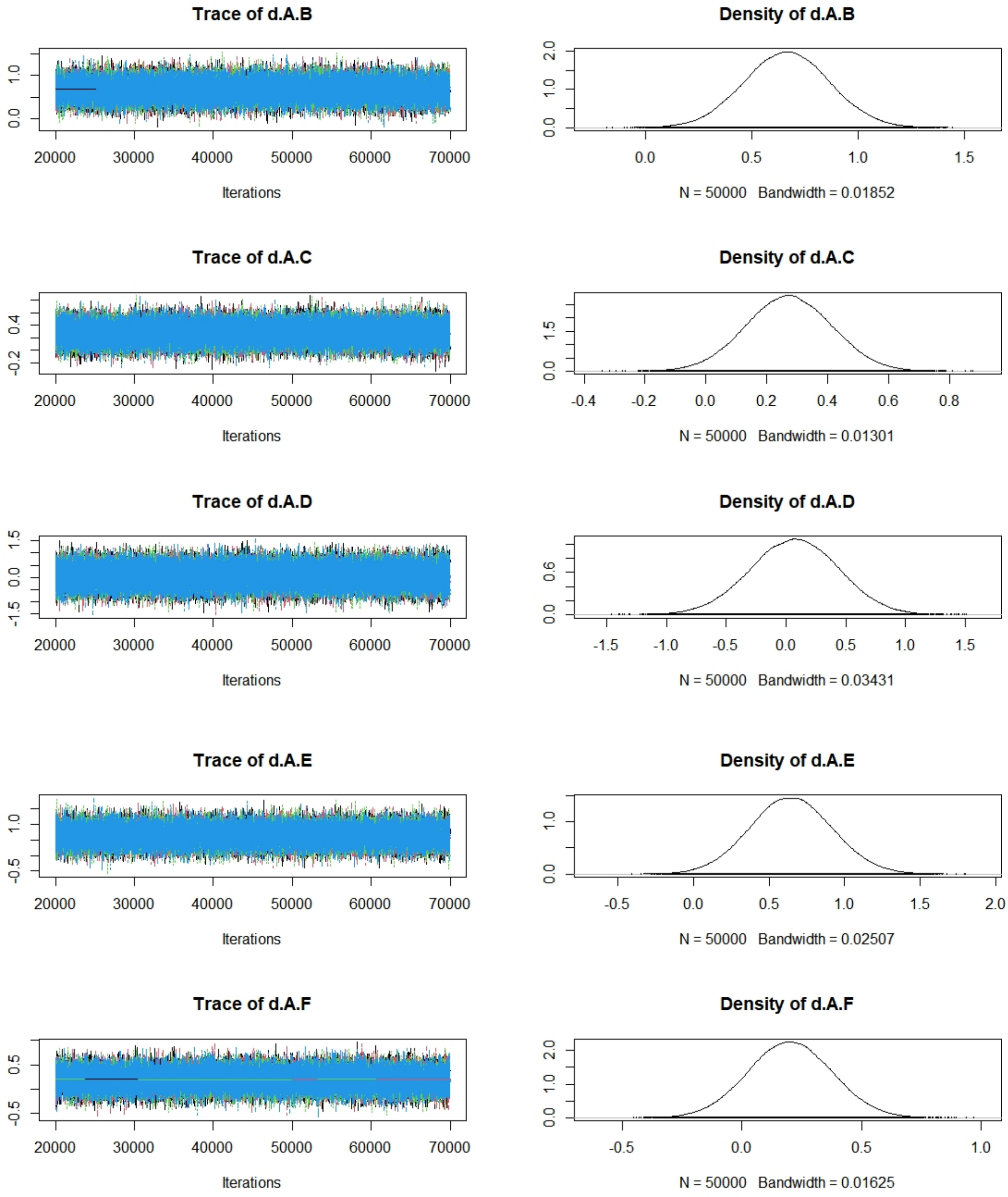

There are no specific patterns and the chains are entangled, it is considered that the convergence is good. A: Endovascular Treatment; B: Endovascular Treatment plus 0.225 mg/kg Alteplase; C: Endovascular Treatment plus 0.0625 mg/kg Tenecteplase; D: Endovascular Treatment plus 0.0313 mg/kg Tenecteplase; E: Endovascular Treatment plus 0.125 mg/kg Tenecteplase; F: Endovascular Treatment plus 100,000 IU Urokinase.

(b) Trace plot and density plot for functional independence (mRS 0–2) at 90 days.

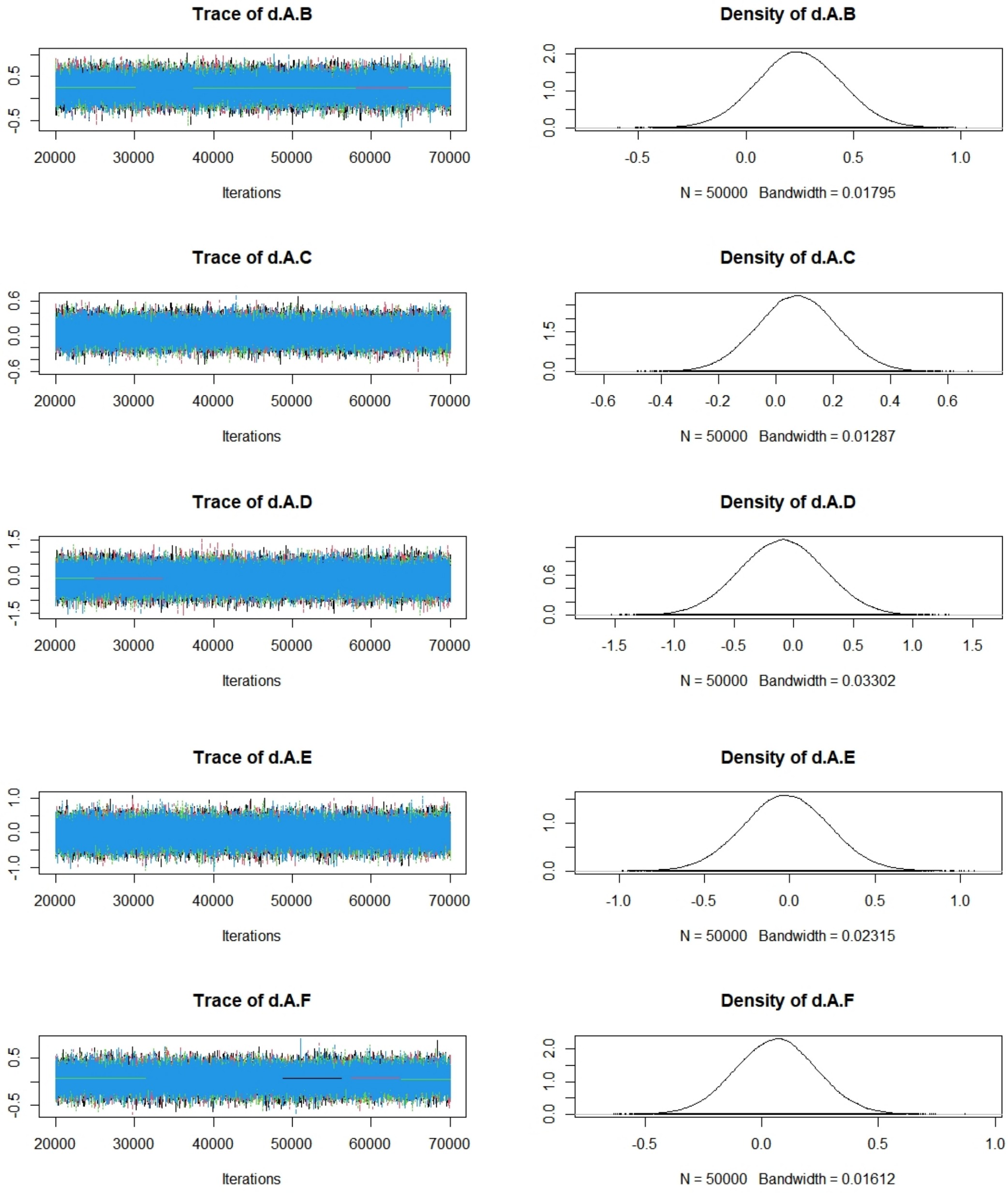

There are no specific patterns and the chains are entangled, it is considered that the convergence is good. A: Endovascular Treatment; B: Endovascular Treatment plus 0.225 mg/kg Alteplase; C: Endovascular Treatment plus 0.0625 mg/kg Tenecteplase; D: Endovascular Treatment plus 0.0313 mg/kg Tenecteplase; E: Endovascular Treatment plus 0.125 mg/kg Tenecteplase; F: Endovascular Treatment plus 100,000 IU Urokinase.

(c) Trace plot and density plot for favorable outcome (mRS 0–3) at 90 days.

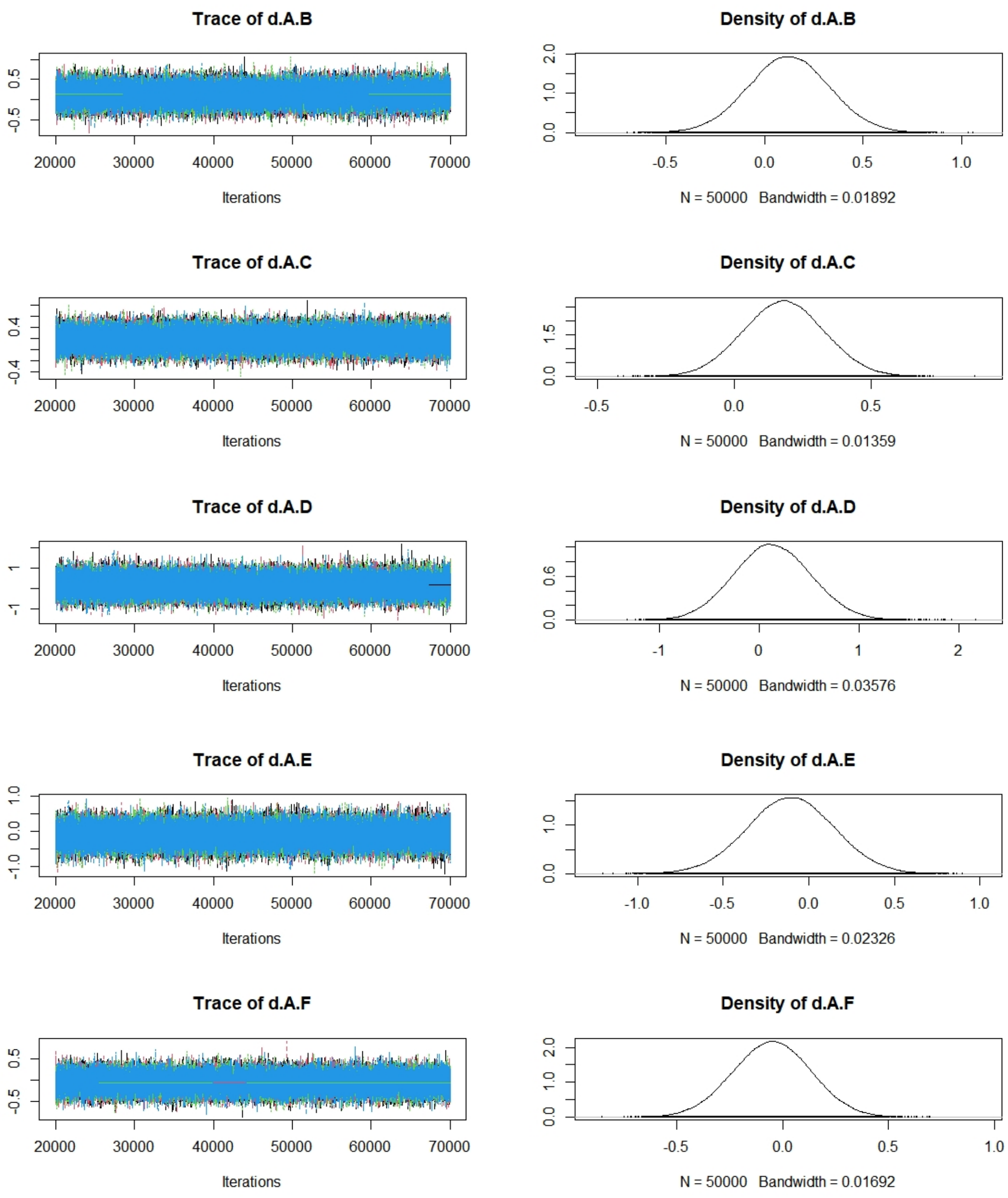

There are no specific patterns and the chains are entangled, it is considered that the convergence is good. A: Endovascular Treatment; B: Endovascular Treatment plus 0.225 mg/kg Alteplase; C: Endovascular Treatment plus 0.0625 mg/kg Tenecteplase; D: Endovascular Treatment plus 0.0313 mg/kg Tenecteplase; E: Endovascular Treatment plus 0.125 mg/kg Tenecteplase; F: Endovascular Treatment plus 100,000 IU Urokinase.

(d) Trace plot and density plot for the death within 90 days.

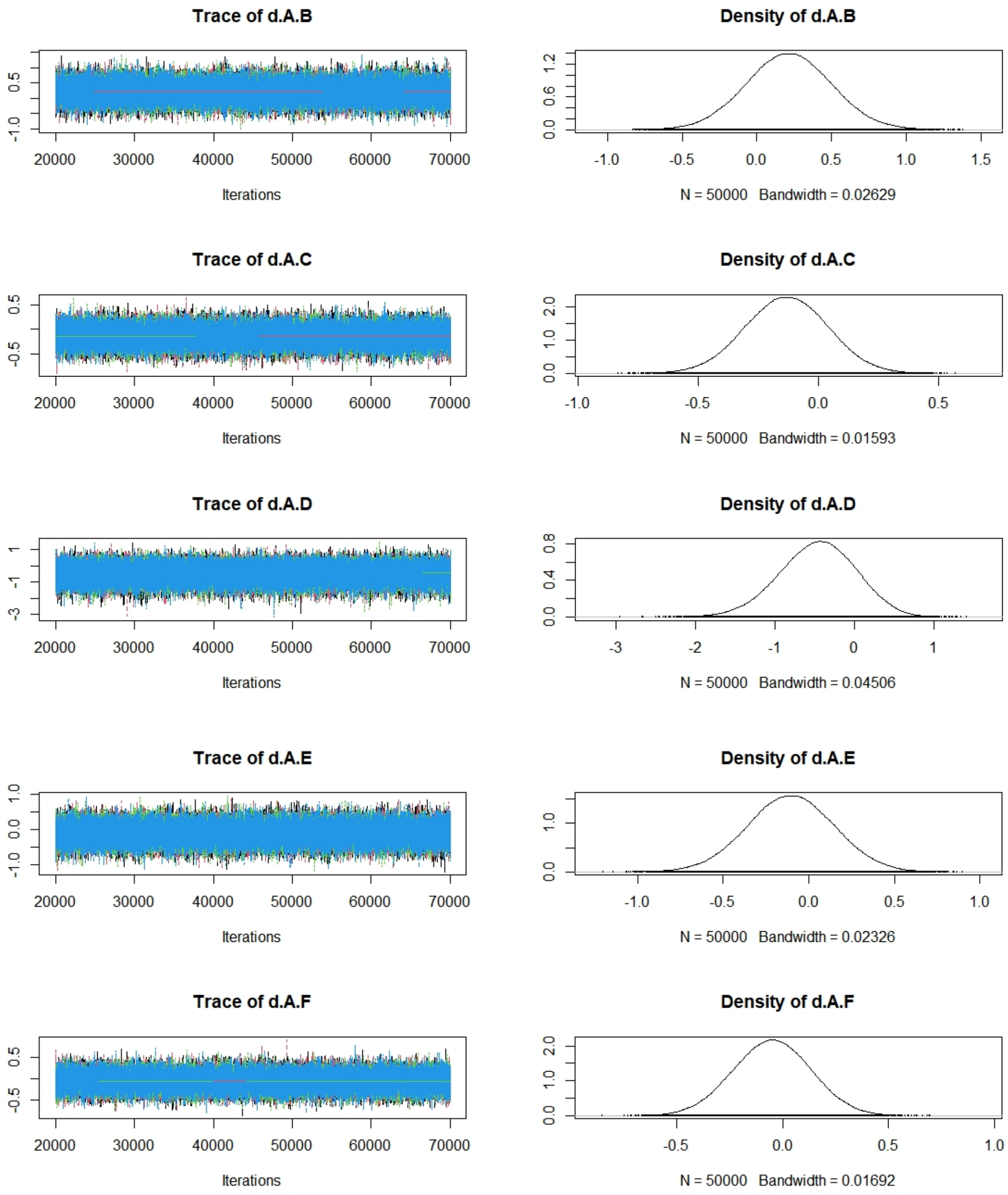

There are no specific patterns and the chains are entangled, it is considered that the convergence is good. A: Endovascular Treatment; B: Endovascular Treatment plus 0.225 mg/kg Alteplase; C: Endovascular Treatment plus 0.0625 mg/kg Tenecteplase; D: Endovascular Treatment plus 0.0313 mg/kg Tenecteplase; E: Endovascular Treatment plus 0.125 mg/kg Tenecteplase; F: Endovascular Treatment plus 100,000 IU Urokinase.

(e) Trace plot and density plot for the symptomatic intracranial hemorrhage.

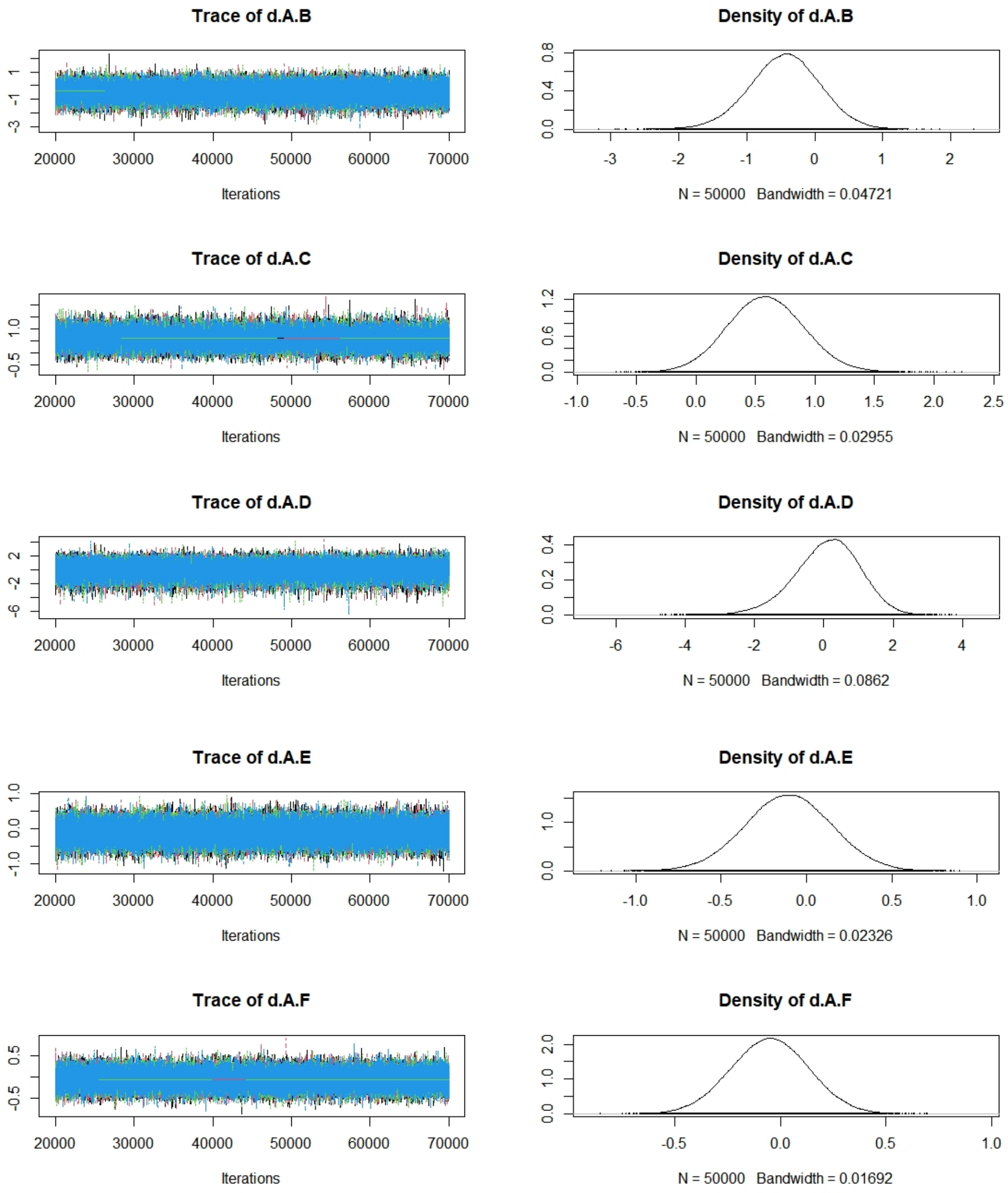

There are no specific patterns and the chains are entangled, it is considered that the convergence is good. A: Endovascular Treatment; B: Endovascular Treatment plus 0.225 mg/kg Alteplase; C: Endovascular Treatment plus 0.0625 mg/kg Tenecteplase; D: Endovascular Treatment plus 0.0313 mg/kg Tenecteplase; E: Endovascular Treatment plus 0.125 mg/kg Tenecteplase; F: Endovascular Treatment plus 100,000 IU Urokinase.

(f) Trace plot and density plot for the any intracranial hemorrhage.

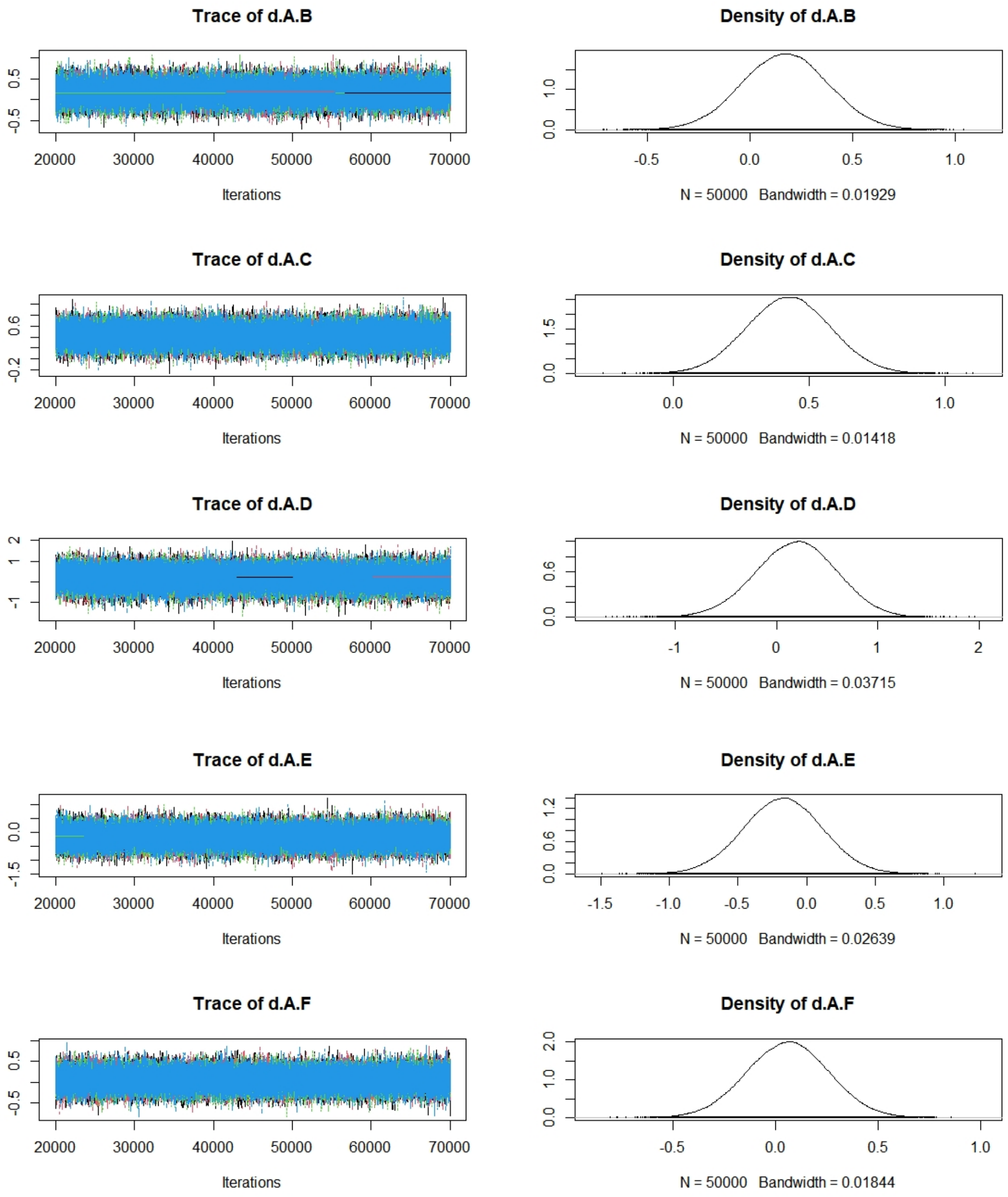

There are no specific patterns and the chains are entangled, it is considered that the convergence is good. A: Endovascular Treatment; B: Endovascular Treatment plus 0.225 mg/kg Alteplase; C: Endovascular Treatment plus 0.0625 mg/kg Tenecteplase; D: Endovascular Treatment plus 0.0313 mg/kg Tenecteplase; E: Endovascular Treatment plus 0.125 mg/kg Tenecteplase; F: Endovascular Treatment plus 100,000 IU Urokinase.

Figure S3. Cochrane risk of bias assessments for each included study.

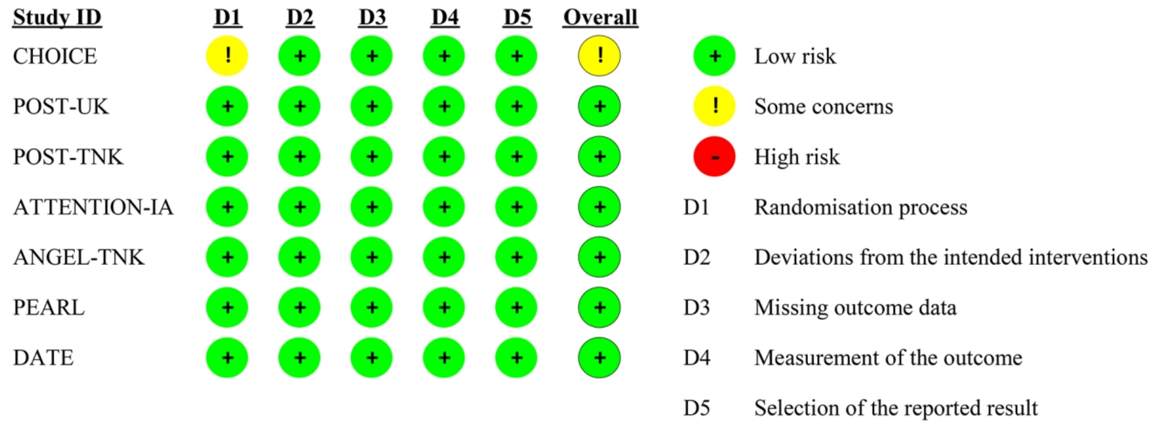

Figure S4. Risk of bias graph.

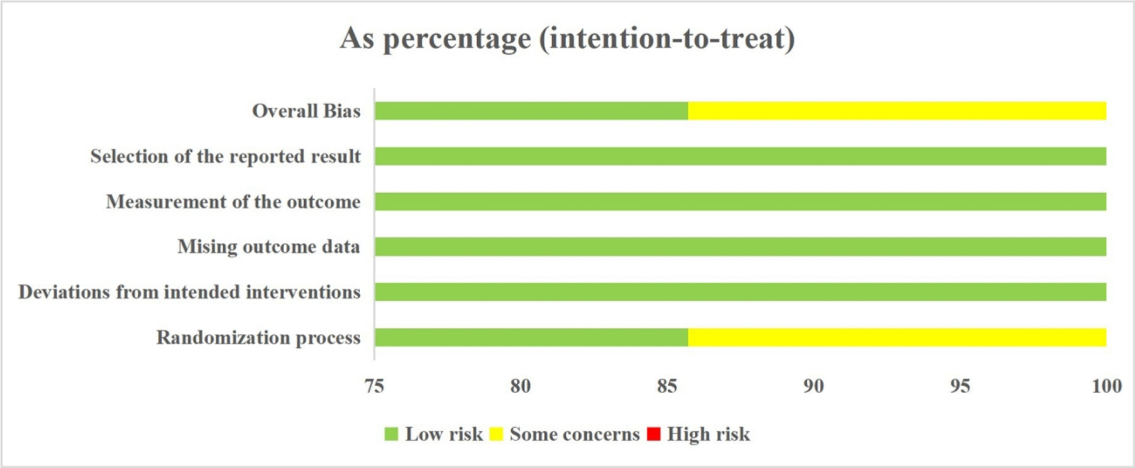

Figure S5. Results of the heterogeneity analysis.

(a) Excellent outcome (mRS 0–1) at 90 days

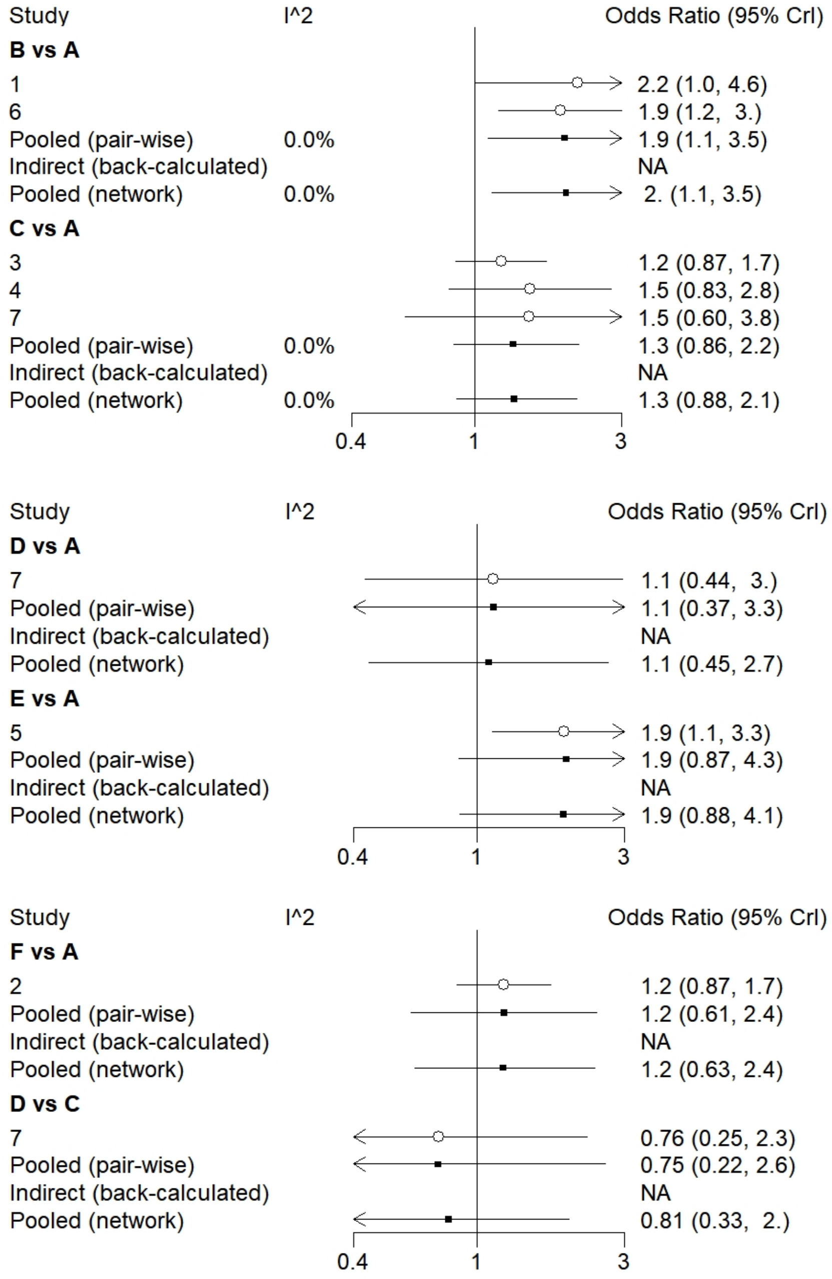

Low I^2 values were observed across all pairwise and network meta-analyses, suggesting acceptable heterogeneity. A: Endovascular Treatment; B: Endovascular Treatment plus 0.225 mg/kg Alteplase; C: Endovascular Treatment plus 0.0625 mg/kg Tenecteplase; D: Endovascular Treatment plus 0.0313 mg/kg Tenecteplase; E: Endovascular Treatment plus 0.125 mg/kg Tenecteplase; F: Endovascular Treatment plus 100,000 IU Urokinase.

(b) Functional independence (mRS 0–2) at 90 days

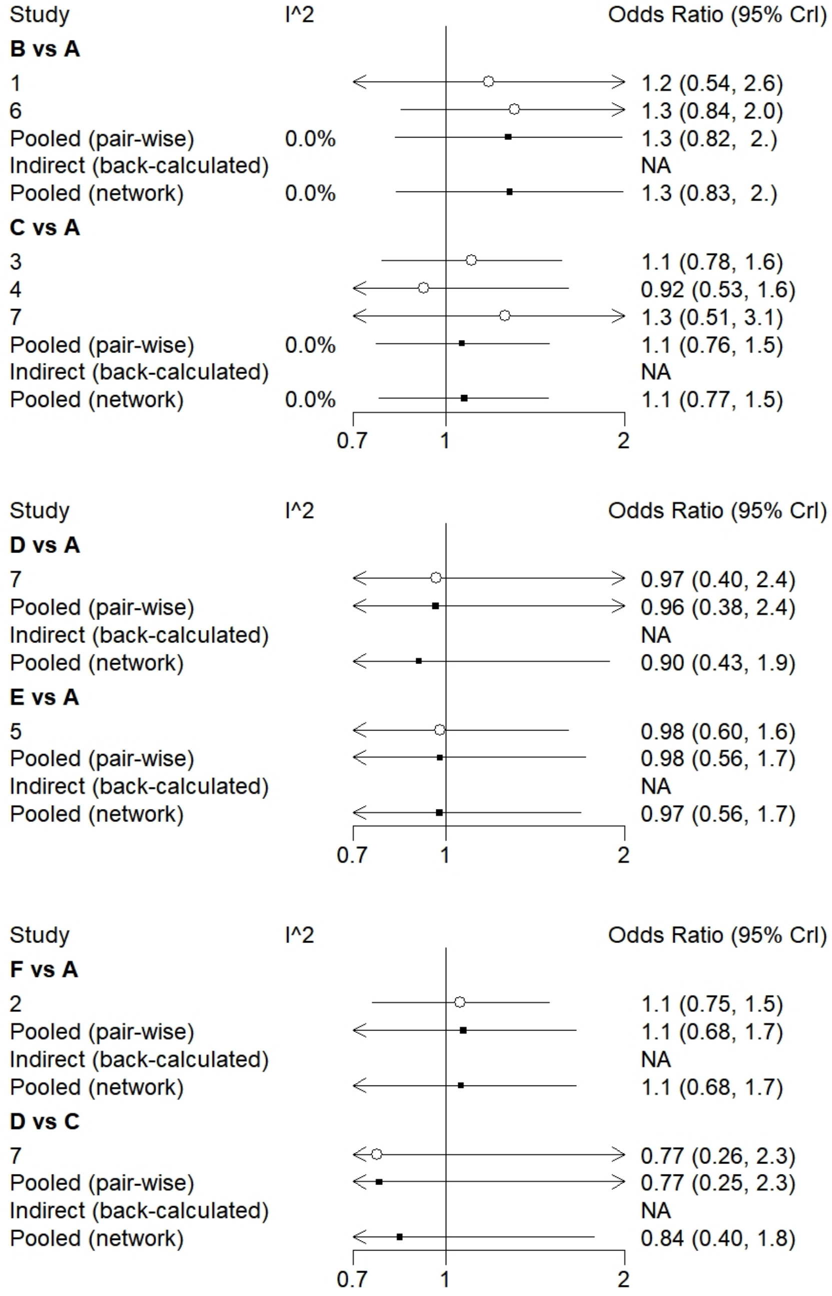

Low I^2 values were observed across all pairwise and network meta-analyses, suggesting acceptable heterogeneity. A: Endovascular Treatment; B: Endovascular Treatment plus 0.225 mg/kg Alteplase; C: Endovascular Treatment plus 0.0625 mg/kg Tenecteplase; D: Endovascular Treatment plus 0.0313 mg/kg Tenecteplase; E: Endovascular Treatment plus 0.125 mg/kg Tenecteplase; F: Endovascular Treatment plus 100,000 IU Urokinase.

(c) Favorable outcome (mRS 0–3) at 90 days

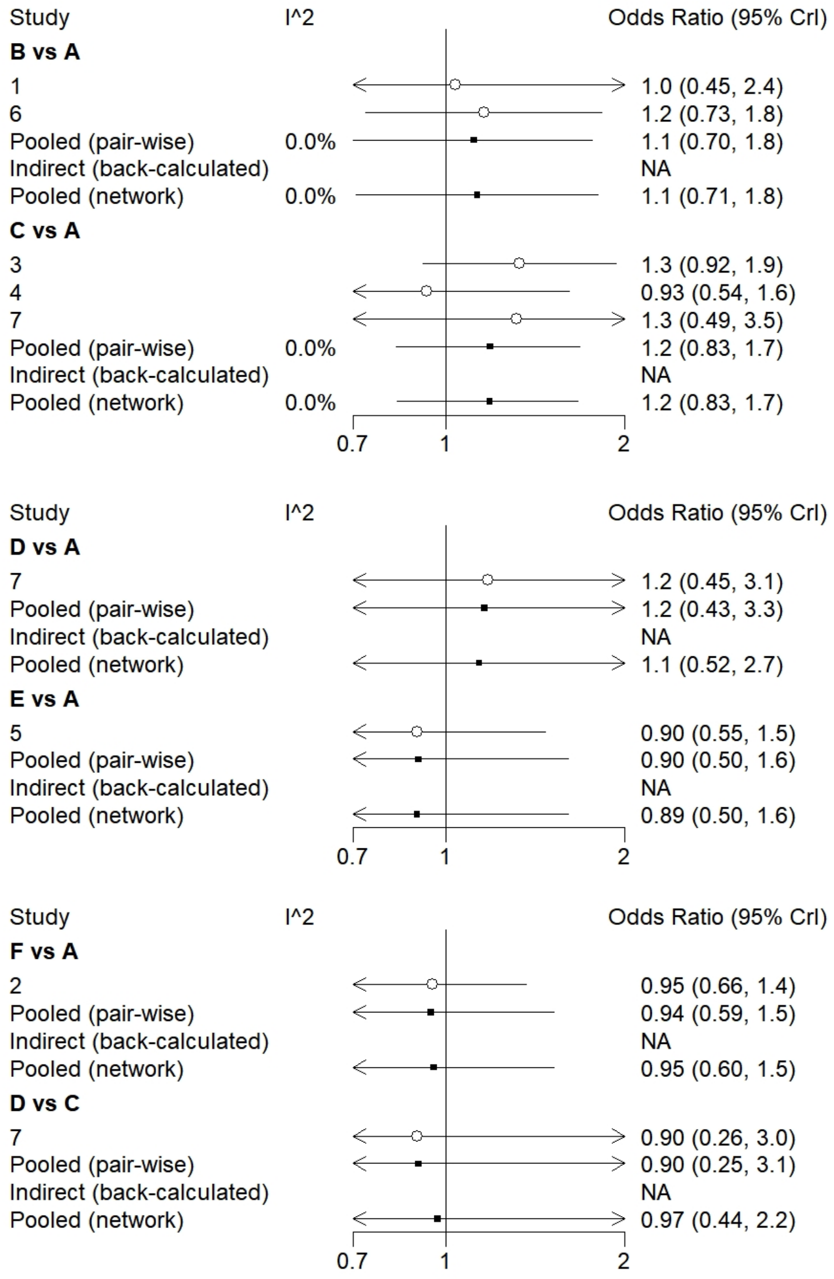

Low I^2 values were observed across all pairwise and network meta-analyses, suggesting acceptable heterogeneity. A: Endovascular Treatment; B: Endovascular Treatment plus 0.225 mg/kg Alteplase; C: Endovascular Treatment plus 0.0625 mg/kg Tenecteplase; D: Endovascular Treatment plus 0.0313 mg/kg Tenecteplase; E: Endovascular Treatment plus 0.125 mg/kg Tenecteplase; F: Endovascular Treatment plus 100,000 IU Urokinase.

(d) Death within 90 days

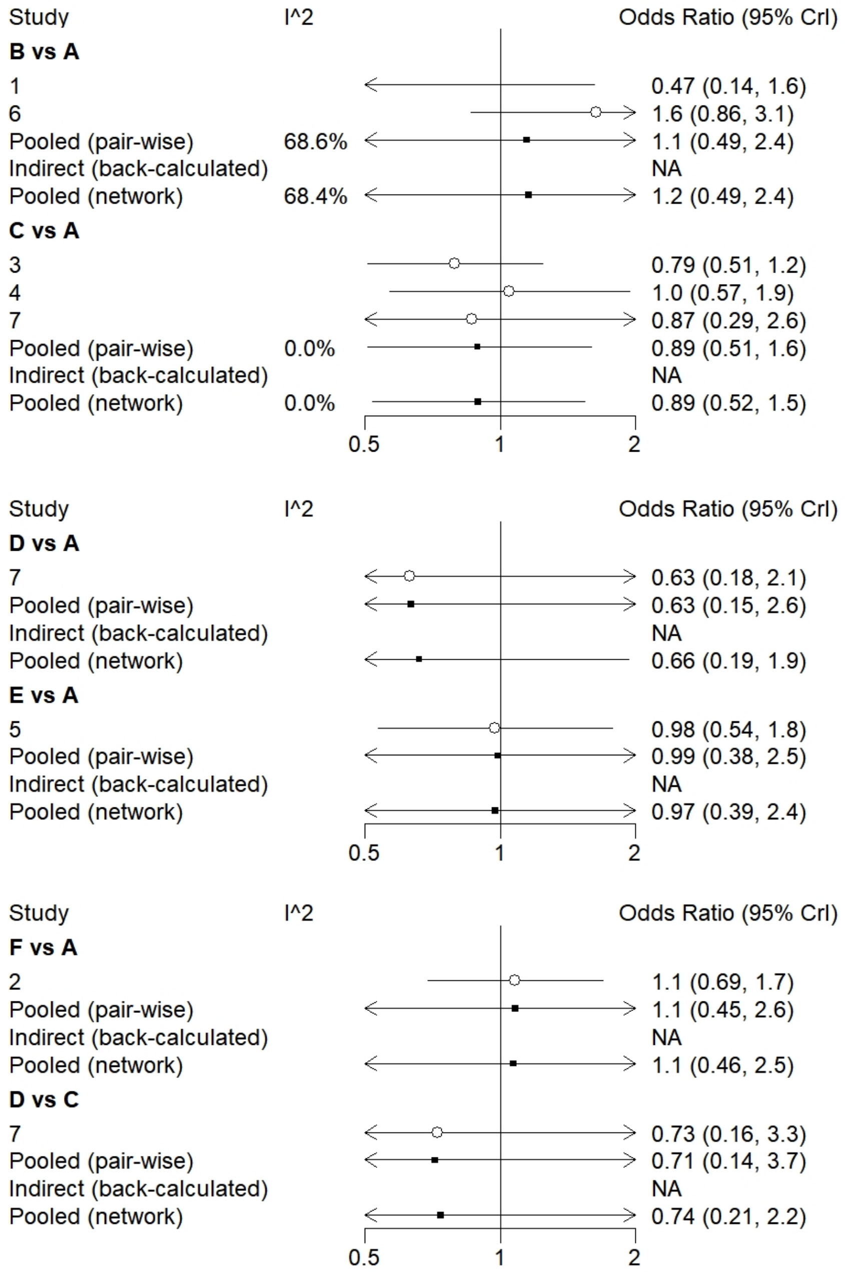

Low heterogeneity was observed in all comparisons except for one pairwise contrasts (B vs. A), which did not affect the primary conclusions. A: Endovascular Treatment; B: Endovascular Treatment plus 0.225 mg/kg Alteplase; C: Endovascular Treatment plus 0.0625 mg/kg Tenecteplase; D: Endovascular Treatment plus 0.0313 mg/kg Tenecteplase; E: Endovascular Treatment plus 0.125 mg/kg Tenecteplase; F: Endovascular Treatment plus 100,000 IU Urokinase.

(e) Symptomatic intracranial hemorrhage

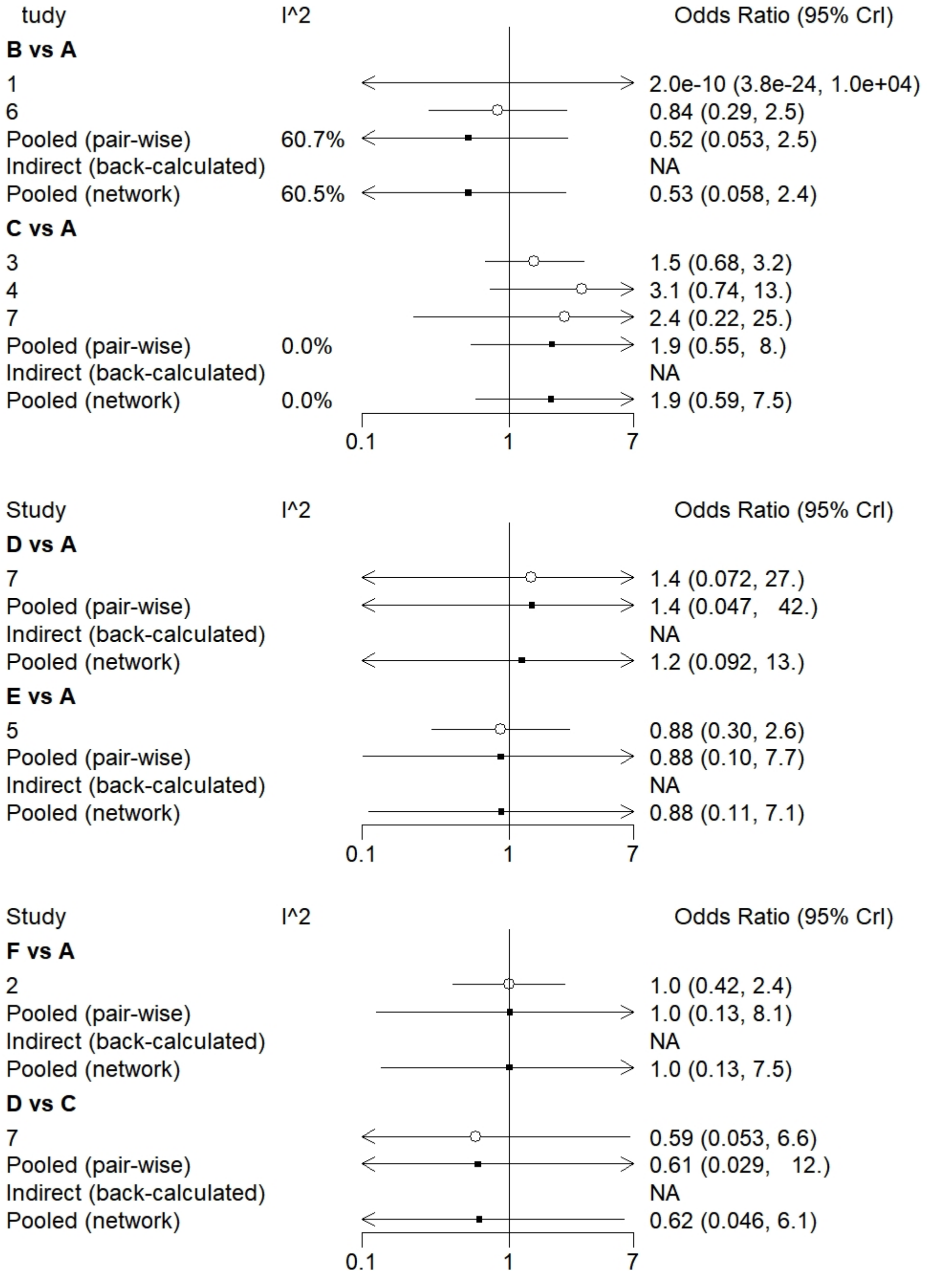

Low heterogeneity was observed in all comparisons except for one pairwise contrasts (B vs. A), which did not affect the primary conclusions. A: Endovascular Treatment; B: Endovascular Treatment plus 0.225 mg/kg Alteplase; C: Endovascular Treatment plus 0.0625 mg/kg Tenecteplase; D: Endovascular Treatment plus 0.0313 mg/kg Tenecteplase; E: Endovascular Treatment plus 0.125 mg/kg Tenecteplase; F: Endovascular Treatment plus 100,000 IU Urokinase.

(f) Any intracranial hemorrhage

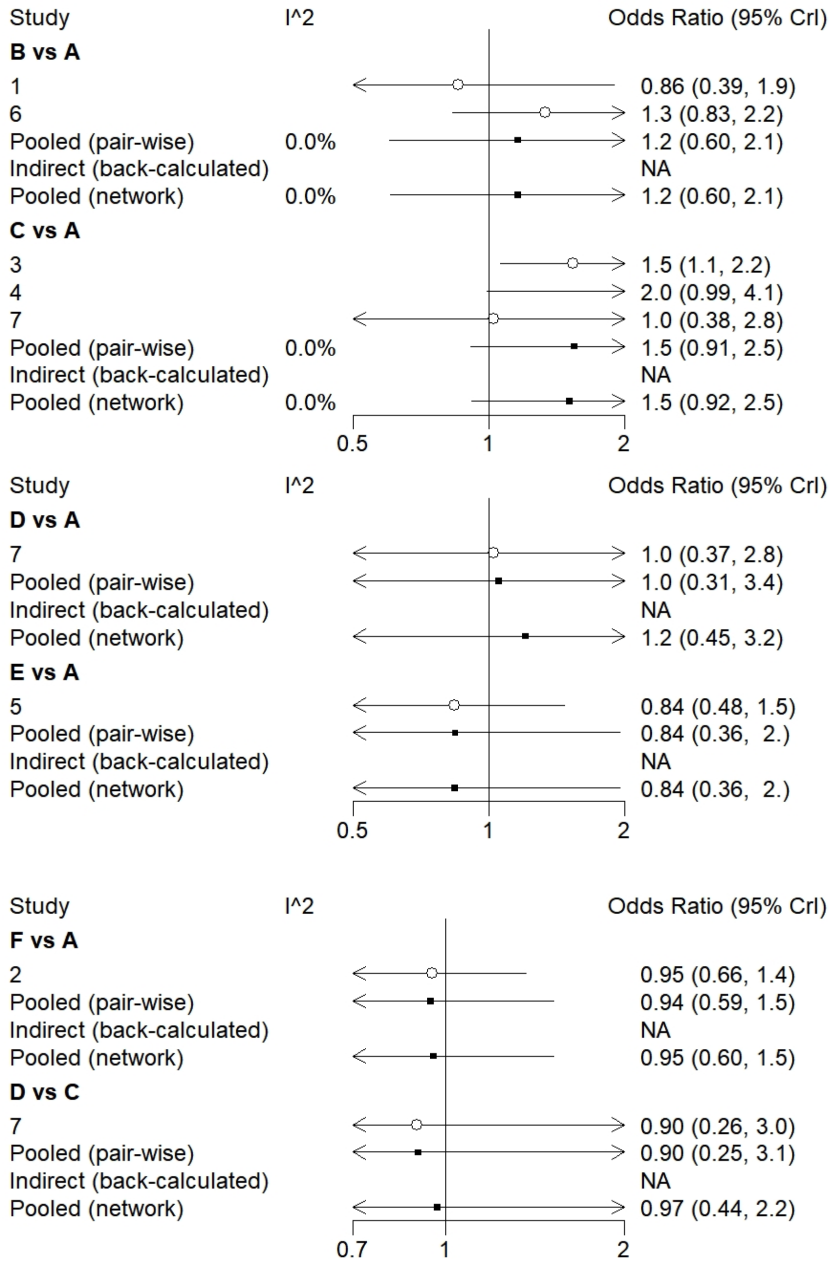

Low I^2 values were observed across all pairwise and network meta-analyses, suggesting acceptable heterogeneity. A: Endovascular Treatment; B: Endovascular Treatment plus 0.225 mg/kg Alteplase; C: Endovascular Treatment plus 0.0625 mg/kg Tenecteplase; D: Endovascular Treatment plus 0.0313 mg/kg Tenecteplase; E: Endovascular Treatment plus 0.125 mg/kg Tenecteplase; F: Endovascular Treatment plus 100,000 IU Urokinase.

Figure S6. Comparison-adjusted funnel plots for publication bias.

(a) Excellent outcome (mRS 0–1) at 90 days

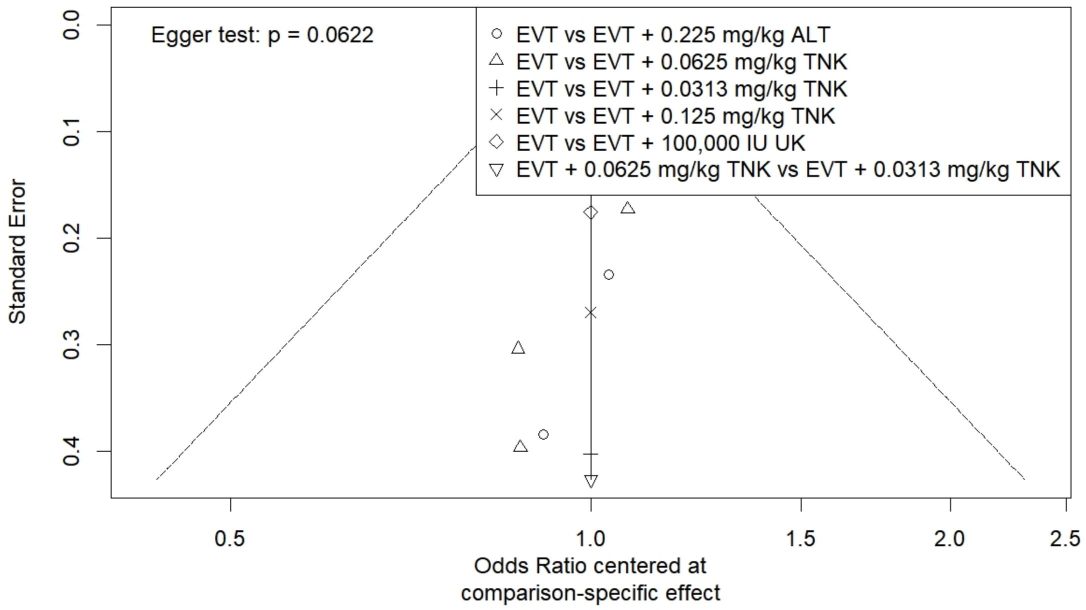

(b) Functional independence (mRS 0–2) at 90 days

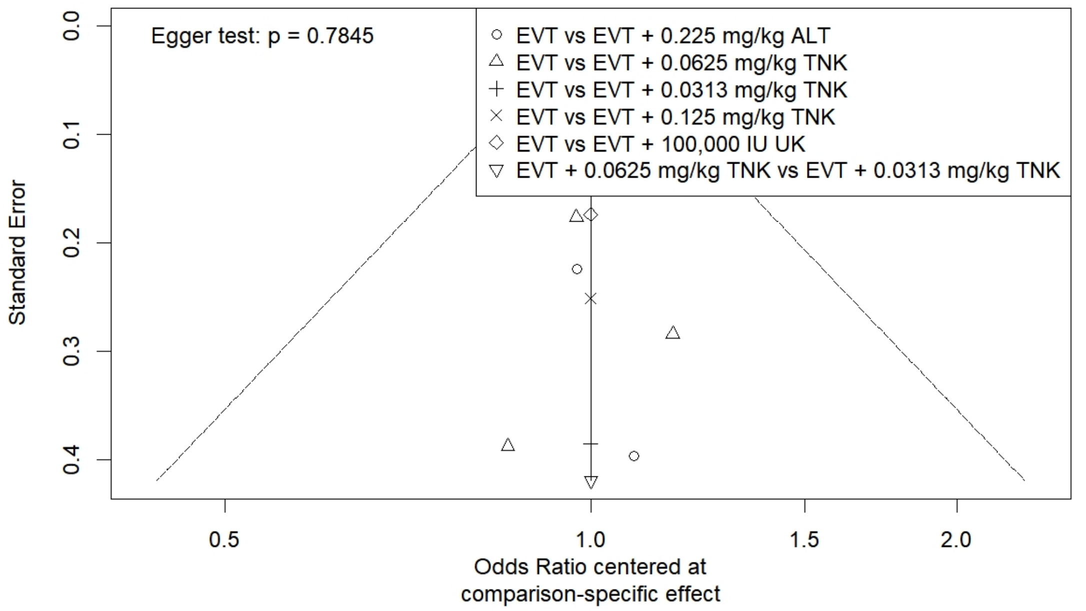

(c) Favorable outcome (mRS 0–3) at 90 days

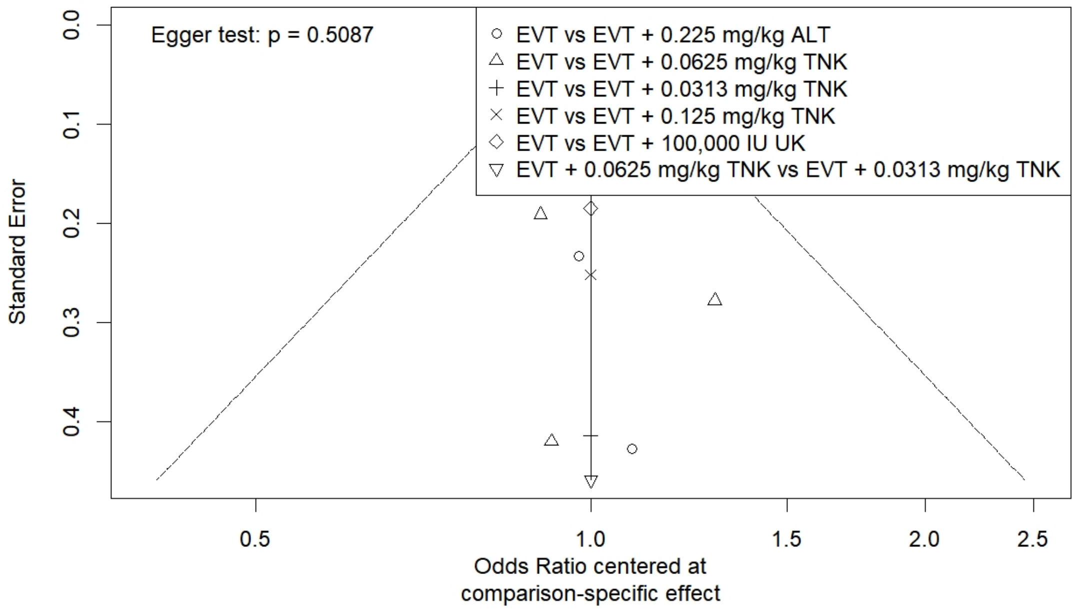

(d) Death within 90 days

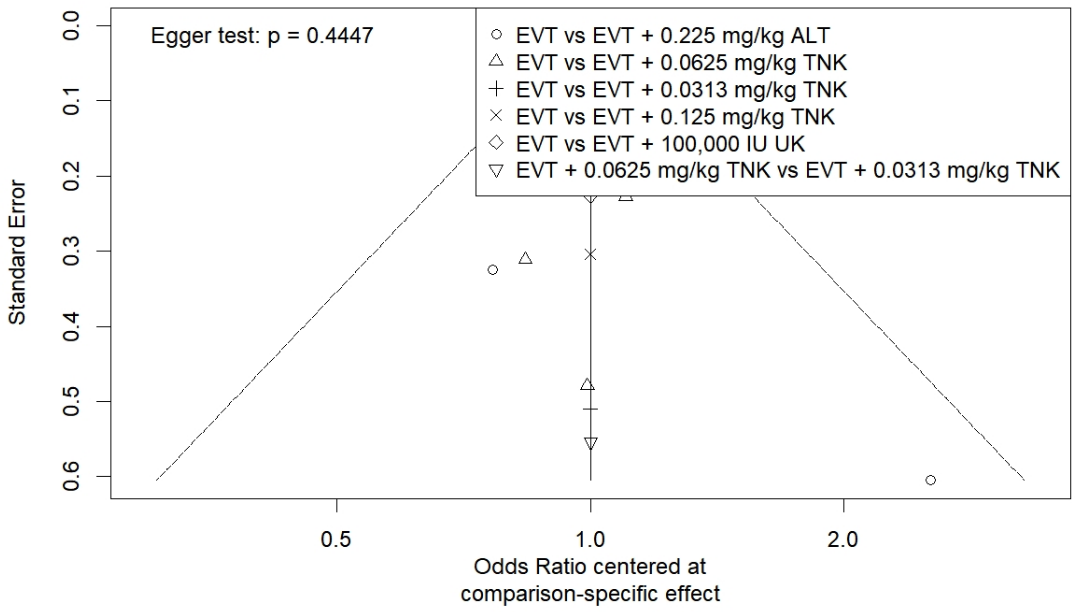

(e) Symptomatic intracranial hemorrhage

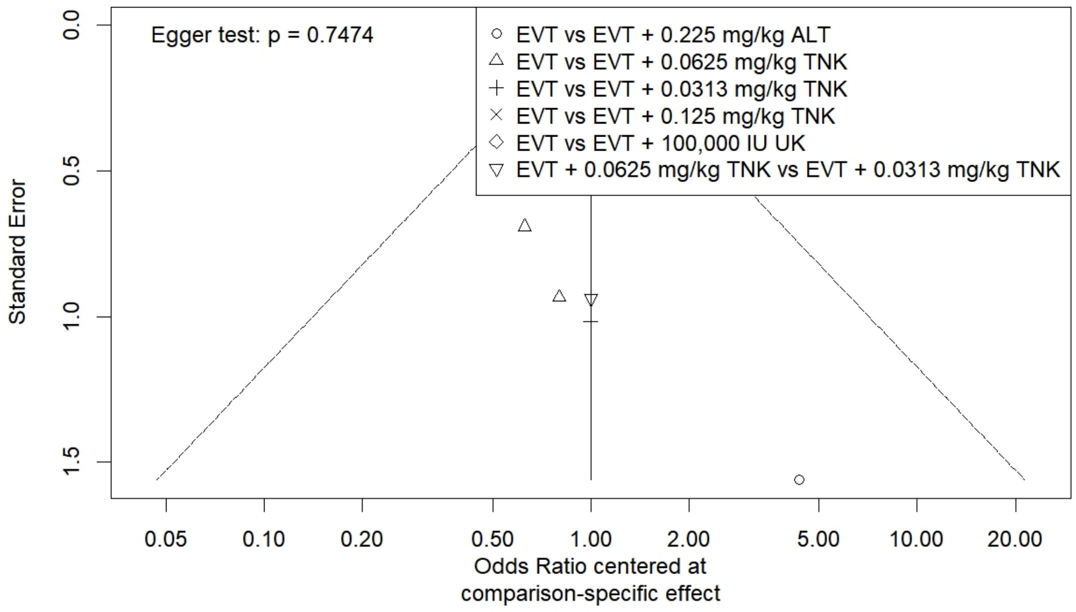

(f) Any intracranial hemorrhage

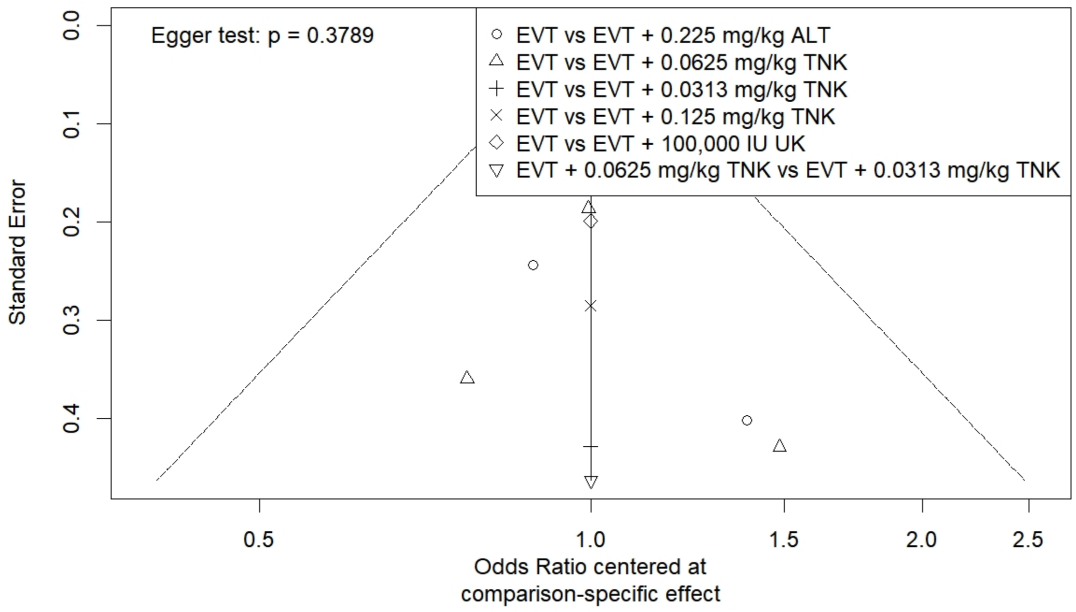

Table S1. Model fit details including the random effect (RE) compared and the fixed effect (FE) model.

	**Deviance**	**Unconstrained data points**	**pD**	**DIC**	**Ratio**	**I^2**
mRS 0-1 RE	13.22	15	12.97	26.19	0.88	0%
mRS 0-1 FE	12.65	15	12.06	24.71	0.84	0%
mRS 0-2 RE	12.93	15	12.54	25.47	0.86	0%
mRS 0-2 FE	12.55	15	12.01	24.56	0.84	0%
mRS 0-3 RE	13.25	15	12.48	25.73	0.88	0%
mRS 0-3 FE	13.27	15	12.02	25.29	0.88	0%
Mortality RE	15.12	15	13.30	28.41	1.01	7%
Mortality FE	15.72	15	12.11	27.83	1.05	11%
sICH RE	15.39	15	13.40	28.78	1.03	9%
sICH FE	15.66	15	12.35	28.01	1.04	11%
aICH RE	14.35	15	13.22	27.57	0.96	2%
aICH FE	14.35	15	12.07	26.42	0.96	2%

Between-study variance (τ^2) was estimated as 0.211, 0.117, 0.127, 0.286, 0.689, and 0.246 for mRS 0–1, mRS 0–2, mRS 0–3, Mortality, sICH, and aICH, respectively. These τ^2 values indicate that variability between studies was generally low, supporting the appropriateness of a fixed-effect specification. As shown in Table S1, heterogeneity across all outcomes was minimal (I^2 = 0–11%). Fixed-effect models consistently yielded lower DIC values than random-effects models, while random-effects models increased model complexity (higher pD) without improving fit, further supporting the selection of fixed-effect models as the most parsimonious and reliable approach. Taken together, minimal I^2, small-to-moderate τ^2, and model fit indices demonstrate that the evidence network is sufficiently homogeneous to justify fixed-effect modeling. mRS 0–1: Excellent outcome at 90 days; mRS 0–2: Functional independence at 90 days; mRS 0–3: Favorable outcome at 90 days; Mortality: Death within 90 days; sICH: Symptomatic intracranial hemorrhage; aICH: Any intracranial hemorrhage.

Table S2. CINeMA Assessment.

(a) CINeMA Assessment Results for excellent outcome (mRS 0–1) at 90 days.

Comparison	Number of studies	Within-study bias	Reporting bias	Indirectness	Imprecision	Heterogeneity	Incoherence	Confidence rating	Reason(s) for downgrading
Mixed evidence									
EVT:EVT+ALT0.225	2	No concerns	Low risk	No concerns	No concerns	Some concerns	No concerns	Moderate	["Heterogeneity"]
EVT:EVT+TNK0.0313	1	No concerns	Low risk	No concerns	Major concerns	No concerns	No concerns	Low	["Imprecision"]
EVT:EVT+TNK0.0625	3	No concerns	Low risk	No concerns	Some concerns	No concerns	No concerns	Moderate	["Imprecision"]
EVT:EVT+TNK0.125	1	No concerns	Low risk	No concerns	No concerns	Major concerns	No concerns	Low	["Heterogeneity"]
EVT:EVT+UK100000IU	1	No concerns	Low risk	No concerns	Some concerns	Some concerns	No concerns	Low	["Imprecision","Heterogeneity"]
EVT+TNK0.0313:EVT+TNK0.0625	1	No concerns	Low risk	No concerns	Major concerns	No concerns	No concerns	Low	["Imprecision"]
Indirected evidence									
EVT+ALT0.225:EVT+TNK0.0313	0	No concerns	Low risk	No concerns	Some concerns	Some concerns	No concerns	Low	["Imprecision","Heterogeneity"]
EVT+ALT0.225:EVT+TNK0.0625	0	No concerns	Low risk	No concerns	Some concerns	Some concerns	No concerns	Low	["Imprecision","Heterogeneity"]
EVT+ALT0.225:EVT+TNK0.125	0	No concerns	Low risk	No concerns	Major concerns	No concerns	No concerns	Low	["Imprecision"]
EVT+ALT0.225:EVT+UK100000IU	0	No concerns	Low risk	No concerns	Some concerns	Some concerns	No concerns	Low	["Imprecision","Heterogeneity"]
EVT+TNK0.0313:EVT+TNK0.125	0	No concerns	Low risk	No concerns	Some concerns	Some concerns	No concerns	Low	["Imprecision","Heterogeneity"]
EVT+TNK0.0313:EVT+UK100000IU	0	No concerns	Low risk	No concerns	Major concerns	No concerns	No concerns	Low	["Imprecision"]
EVT+TNK0.0625:EVT+TNK0.125	0	No concerns	Low risk	No concerns	Some concerns	Some concerns	No concerns	Low	["Imprecision","Heterogeneity"]
EVT+TNK0.0625:EVT+UK100000IU	0	No concerns	Low risk	No concerns	Major concerns	No concerns	No concerns	Low	["Imprecision"]
EVT+TNK0.125:EVT+UK100000IU	0	No concerns	Low risk	No concerns	Some concerns	Some concerns	No concerns	Low	["Imprecision","Heterogeneity"]

The confidence rating for all comparisons was Low or Moderate, with the minimal clinically important difference pre-defined as an odds ratio interval of 0.70 to 1.43 for imprecision.

(b) CINeMA Assessment Results for functional independence (mRS 0–2) at 90 days.

Comparison	Number of studies	Within-study bias	Reporting bias	Indirectness	Imprecision	Heterogeneity	Incoherence	Confidence rating	Reason(s) for downgrading
Mixed evidence									
EVT:EVT+ALT0.225	2	No concerns	Low risk	No concerns	Some concerns	Some concerns	No concerns	Low	["Imprecision","Heterogeneity"]
EVT:EVT+TNK0.0313	1	No concerns	Low risk	No concerns	Major concerns	No concerns	No concerns	Low	["Imprecision"]
EVT:EVT+TNK0.0625	3	No concerns	Low risk	No concerns	No concerns	Major concerns	No concerns	Low	["Heterogeneity"]
EVT:EVT+TNK0.125	1	No concerns	Low risk	No concerns	Major concerns	No concerns	No concerns	Low	["Imprecision"]
EVT:EVT+UK100000IU	1	No concerns	Low risk	No concerns	Some concerns	Some concerns	No concerns	Low	["Imprecision","Heterogeneity"]
EVT+TNK0.0313:EVT+TNK0.0625	1	No concerns	Low risk	No concerns	Major concerns	No concerns	No concerns	Low	["Imprecision"]
Indirected evidence									
EVT+ALT0.225:EVT+TNK0.0313	0	No concerns	Low risk	No concerns	Major concerns	No concerns	No concerns	Low	["Imprecision"]
EVT+ALT0.225:EVT+TNK0.0625	0	No concerns	Low risk	No concerns	Some concerns	Some concerns	No concerns	Low	["Imprecision","Heterogeneity"]
EVT+ALT0.225:EVT+TNK0.125	0	No concerns	Low risk	No concerns	Some concerns	Some concerns	No concerns	Low	["Imprecision","Heterogeneity"]
EVT+ALT0.225:EVT+UK100000IU	0	No concerns	Low risk	No concerns	Some concerns	Some concerns	No concerns	Low	["Imprecision","Heterogeneity"]
EVT+TNK0.0313:EVT+TNK0.125	0	No concerns	Low risk	No concerns	Major concerns	No concerns	No concerns	Low	["Imprecision"]
EVT+TNK0.0313:EVT+UK100000IU	0	No concerns	Low risk	No concerns	Major concerns	No concerns	No concerns	Low	["Imprecision"]
EVT+TNK0.0625:EVT+TNK0.125	0	No concerns	Low risk	No concerns	Major concerns	No concerns	No concerns	Low	["Imprecision"]
EVT+TNK0.0625:EVT+UK100000IU	0	No concerns	Low risk	No concerns	Major concerns	No concerns	No concerns	Low	["Imprecision"]
EVT+TNK0.125:EVT+UK100000IU	0	No concerns	Low risk	No concerns	Major concerns	No concerns	No concerns	Low	["Imprecision"]

The confidence rating for all comparisons was Low, suggesting that the results for this outcome should be interpreted with caution due to the inherent limitations of the evidence network; the minimal clinically important difference was pre-defined as an odds ratio interval of 0.70 to 1.43 for the assessment of imprecision.

(c) CINeMA Assessment Results for favorable outcome (mRS 0–3) at 90 days.

Comparison	Number of studies	Within-study bias	Reporting bias	Indirectness	Imprecision	Heterogeneity	Incoherence	Confidence rating	Reason(s) for downgrading
Mixed evidence									
EVT:EVT+ALT0.225	2	No concerns	Low risk	No concerns	Some concerns	Some concerns	No concerns	Low	["Imprecision","Heterogeneity"]
EVT:EVT+TNK0.0313	1	No concerns	Low risk	No concerns	Major concerns	No concerns	No concerns	Low	["Imprecision"]
EVT:EVT+TNK0.0625	3	No concerns	Low risk	No concerns	Some concerns	Some concerns	No concerns	Low	["Imprecision","Heterogeneity"]
EVT:EVT+TNK0.125	1	No concerns	Low risk	No concerns	Major concerns	No concerns	No concerns	Low	["Imprecision"]
EVT:EVT+UK100000IU	1	No concerns	Low risk	No concerns	Some concerns	Some concerns	No concerns	Low	["Imprecision","Heterogeneity"]
EVT+TNK0.0313:EVT+TNK0.0625	1	No concerns	Low risk	No concerns	Major concerns	No concerns	No concerns	Low	["Imprecision"]
Indirected evidence									
EVT+ALT0.225:EVT+TNK0.0313	0	No concerns	Low risk	No concerns	Major concerns	No concerns	No concerns	Low	["Imprecision"]
EVT+ALT0.225:EVT+TNK0.0625	0	No concerns	Low risk	No concerns	Major concerns	No concerns	No concerns	Low	["Imprecision"]
EVT+ALT0.225:EVT+TNK0.125	0	No concerns	Low risk	No concerns	Major concerns	No concerns	No concerns	Low	["Imprecision"]
EVT+ALT0.225:EVT+UK100000IU	0	No concerns	Low risk	No concerns	Major concerns	No concerns	No concerns	Low	["Imprecision"]
EVT+TNK0.0313:EVT+TNK0.125	0	No concerns	Low risk	No concerns	Major concerns	No concerns	No concerns	Low	["Imprecision"]
EVT+TNK0.0313:EVT+UK100000IU	0	No concerns	Low risk	No concerns	Major concerns	No concerns	No concerns	Low	["Imprecision"]
EVT+TNK0.0625:EVT+TNK0.125	0	No concerns	Low risk	No concerns	Some concerns	Some concerns	No concerns	Low	["Imprecision","Heterogeneity"]
EVT+TNK0.0625:EVT+UK100000IU	0	No concerns	Low risk	No concerns	Some concerns	Some concerns	No concerns	Low	["Imprecision","Heterogeneity"]
EVT+TNK0.125:EVT+UK100000IU	0	No concerns	Low risk	No concerns	Major concerns	No concerns	No concerns	Low	["Imprecision"]

The confidence rating for all comparisons was Low, suggesting that the results for this outcome should be interpreted with caution due to the inherent limitations of the evidence network; the minimal clinically important difference was pre-defined as an odds ratio interval of 0.70 to 1.43 for the assessment of imprecision.

(d) CINeMA Assessment Results for death within 90 days.

Comparison	Number of studies	Within-study bias	Reporting bias	Indirectness	Imprecision	Heterogeneity	Incoherence	Confidence rating	Reason(s) for downgrading
Mixed evidence									
EVT:EVT+ALT0.225	2	No concerns	Low risk	No concerns	Some concerns	Some concerns	No concerns	Low	["Imprecision","Heterogeneity"]
EVT:EVT+TNK0.0313	1	No concerns	Low risk	No concerns	Major concerns	No concerns	No concerns	Low	["Imprecision"]
EVT:EVT+TNK0.0625	3	No concerns	Low risk	No concerns	Some concerns	Some concerns	No concerns	Low	["Imprecision","Heterogeneity"]
EVT:EVT+TNK0.125	1	No concerns	Low risk	No concerns	Major concerns	No concerns	No concerns	Low	["Imprecision"]
EVT:EVT+UK100000IU	1	No concerns	Low risk	No concerns	Major concerns	No concerns	No concerns	Low	["Imprecision","Heterogeneity"]
EVT+TNK0.0313:EVT+TNK0.0625	1	No concerns	Low risk	No concerns	Major concerns	No concerns	No concerns	Low	["Imprecision"]
Indirected evidence									
EVT+ALT0.225:EVT+TNK0.0313	0	No concerns	Low risk	No concerns	Major concerns	No concerns	No concerns	Low	["Imprecision"]
EVT+ALT0.225:EVT+TNK0.0625	0	No concerns	Low risk	No concerns	Some concerns	Some concerns	No concerns	Low	["Imprecision","Heterogeneity"]
EVT+ALT0.225:EVT+TNK0.125	0	No concerns	Low risk	No concerns	Major concerns	No concerns	No concerns	Low	["Imprecision"]
EVT+ALT0.225:EVT+UK100000IU	0	No concerns	Low risk	No concerns	Major concerns	No concerns	No concerns	Low	["Imprecision"]
EVT+TNK0.0313:EVT+TNK0.125	0	No concerns	Low risk	No concerns	Major concerns	No concerns	No concerns	Low	["Imprecision"]
EVT+TNK0.0313:EVT+UK100000IU	0	No concerns	Low risk	No concerns	Major concerns	No concerns	No concerns	Low	["Imprecision"]
EVT+TNK0.0625:EVT+TNK0.125	0	No concerns	Low risk	No concerns	Major concerns	No concerns	No concerns	Low	["Imprecision"]
EVT+TNK0.0625:EVT+UK100000IU	0	No concerns	Low risk	No concerns	Some concerns	Some concerns	No concerns	Low	["Imprecision","Heterogeneity"]
EVT+TNK0.125:EVT+UK100000IU	0	No concerns	Low risk	No concerns	Major concerns	No concerns	No concerns	Low	["Imprecision"]

The confidence rating for all comparisons was Low, suggesting that the results for this outcome should be interpreted with caution due to the inherent limitations of the evidence network; the minimal clinically important difference was pre-defined as an odds ratio interval of 0.70 to 1.43 for the assessment of imprecision.

(e) CINeMA Assessment Results for symptomatic intracranial hemorrhage.

Comparison	Number of studies	Within-study bias	Reporting bias	Indirectness	Imprecision	Heterogeneity	Incoherence	Confidence rating	Reason(s) for downgrading
Mixed evidence									
EVT:EVT+ALT0.225	2	No concerns	Low risk	No concerns	Major concerns	No concerns	No concerns	Low	["Imprecision"]
EVT:EVT+TNK0.0313	1	No concerns	Low risk	No concerns	Major concerns	No concerns	No concerns	Low	["Imprecision"]
EVT:EVT+TNK0.0625	3	No concerns	Low risk	No concerns	Some concerns	Some concerns	No concerns	Low	["Imprecision","Heterogeneity"]
EVT:EVT+TNK0.125	1	No concerns	Low risk	No concerns	Major concerns	No concerns	No concerns	Low	["Imprecision"]
EVT:EVT+UK100000IU	1	No concerns	Low risk	No concerns	Major concerns	No concerns	No concerns	Low	["Imprecision"]
EVT+TNK0.0313:EVT+TNK0.0625	1	No concerns	Low risk	No concerns	Major concerns	No concerns	No concerns	Low	["Imprecision"]
Indirected evidence									
EVT+ALT0.225:EVT+TNK0.0313	0	No concerns	Low risk	No concerns	Major concerns	No concerns	No concerns	Low	["Imprecision"]
EVT+ALT0.225:EVT+TNK0.0625	0	No concerns	Low risk	No concerns	Some concerns	Some concerns	No concerns	Low	["Imprecision","Heterogeneity"]
EVT+ALT0.225:EVT+TNK0.125	0	No concerns	Low risk	No concerns	Major concerns	No concerns	No concerns	Low	["Imprecision"]
EVT+ALT0.225:EVT+UK100000IU	0	No concerns	Low risk	No concerns	Major concerns	No concerns	No concerns	Low	["Imprecision"]
EVT+TNK0.0313:EVT+TNK0.125	0	No concerns	Low risk	No concerns	Major concerns	No concerns	No concerns	Low	["Imprecision"]
EVT+TNK0.0313:EVT+UK100000IU	0	No concerns	Low risk	No concerns	Major concerns	No concerns	No concerns	Low	["Imprecision"]
EVT+TNK0.0625:EVT+TNK0.125	0	No concerns	Low risk	No concerns	Major concerns	No concerns	No concerns	Low	["Imprecision"]
EVT+TNK0.0625:EVT+UK100000IU	0	No concerns	Low risk	No concerns	Major concerns	No concerns	No concerns	Low	["Imprecision"]
EVT+TNK0.125:EVT+UK100000IU	0	No concerns	Low risk	No concerns	Major concerns	No concerns	No concerns	Low	["Imprecision"]

The confidence rating for all comparisons was Low, suggesting that the results for this outcome should be interpreted with caution due to the inherent limitations of the evidence network; the minimal clinically important difference was pre-defined as an odds ratio interval of 0.70 to 1.43 for the assessment of imprecision.

(f) CINeMA Assessment Results for any intracranial hemorrhage.

Comparison	Number of studies	Within-study bias	Reporting bias	Indirectness	Imprecision	Heterogeneity	Incoherence	Confidence rating	Reason(s) for downgrading
Mixed evidence									
EVT:EVT+ALT0.225	2	No concerns	Low risk	No concerns	Some concerns	Some concerns	No concerns	Low	["Imprecision","Heterogeneity"]
EVT:EVT+TNK0.0313	1	No concerns	Low risk	No concerns	Major concerns	No concerns	No concerns	Low	["Imprecision"]
EVT:EVT+TNK0.0625	3	No concerns	Low risk	No concerns	No concerns	Some concerns	No concerns	Moderate	["Heterogeneity"]
EVT:EVT+TNK0.125	1	No concerns	Low risk	No concerns	Major concerns	No concerns	No concerns	Low	["Imprecision"]
EVT:EVT+UK100000IU	1	No concerns	Low risk	No concerns	Some concerns	Some concerns	No concerns	Low	["Imprecision","Heterogeneity"]
EVT+TNK0.0313:EVT+TNK0.0625	1	No concerns	Low risk	No concerns	Major concerns	No concerns	No concerns	Low	["Imprecision"]
Indirected evidence									
EVT+ALT0.225:EVT+TNK0.0313	0	No concerns	Low risk	No concerns	Major concerns	No concerns	No concerns	Low	["Imprecision"]
EVT+ALT0.225:EVT+TNK0.0625	0	No concerns	Low risk	No concerns	Some concerns	Some concerns	No concerns	Low	["Imprecision","Heterogeneity"]
EVT+ALT0.225:EVT+TNK0.125	0	No concerns	Low risk	No concerns	Some concerns	Some concerns	No concerns	Low	["Imprecision","Heterogeneity"]
EVT+ALT0.225:EVT+UK100000IU	0	No concerns	Low risk	No concerns	Major concerns	No concerns	No concerns	Low	["Imprecision"]
EVT+TNK0.0313:EVT+TNK0.125	0	No concerns	Low risk	No concerns	Major concerns	No concerns	No concerns	Low	["Imprecision"]
EVT+TNK0.0313:EVT+UK100000IU	0	No concerns	Low risk	No concerns	Major concerns	No concerns	No concerns	Low	["Imprecision"]
EVT+TNK0.0625:EVT+TNK0.125	0	No concerns	Low risk	No concerns	Some concerns	Some concerns	No concerns	Low	["Imprecision","Heterogeneity"]
EVT+TNK0.0625:EVT+UK100000IU	0	No concerns	Low risk	No concerns	Some concerns	Some concerns	No concerns	Low	["Imprecision","Heterogeneity"]
EVT+TNK0.125:EVT+UK100000IU	0	No concerns	Low risk	No concerns	Major concerns	No concerns	No concerns	Low	["Imprecision"]

The confidence rating for all comparisons was Low or Moderate, with the minimal clinically important difference pre-defined as an odds ratio interval of 0.70 to 1.43 for imprecision.

Table S3. Results of subgroup analyses.

(a) Subgroup analyses about the pure subgroup (EVT + IAT).

	**OR (95% CrI)**	**SUCRA**	**Rank**
**Excellent outcome (mRS 0–1) at 90 days**			
Endovascular Treatment		0.161	5
Endovascular Treatment plus 0.0313 mg/kg Tenecteplase	1.06 (0.50, 2.19)	0.334	4
Endovascular Treatment plus 0.0625 mg/kg Tenecteplase	1.27 (0.93, 1.73)	0.567	2
Endovascular Treatment plus 0.125 mg/kg Tenecteplase	**1.91 (1.13, 3.26)**	**0.930**	**1**
Endovascular Treatment plus 100,000 IU Urokinase	1.22 (0.87, 1.73)	0.508	3
**Functional independence (mRS 0–2) at 90 days**			
Endovascular Treatment		0.425	4
Endovascular Treatment plus 0.0313 mg/kg Tenecteplase	0.93 (0.46, 1.87)	0.379	5
Endovascular Treatment plus 0.0625 mg/kg Tenecteplase	1.13 (0.83, 1.55)	**0.697**	**1**
Endovascular Treatment plus 0.125 mg/kg Tenecteplase	0.98 (0.60, 1.61)	0.433	3
Endovascular Treatment plus 100,000 IU Urokinase	1.06 (0.75, 1.49)	0.566	2
**Favorable outcome (mRS 0–3) at 90 days**			
Endovascular Treatment		0.413	3
Endovascular Treatment plus 0.0313 mg/kg Tenecteplase	1.19 (0.56, 2.58)	0.622	2
Endovascular Treatment plus 0.0625 mg/kg Tenecteplase	1.33 (0.94, 1.86)	**0.840**	**1**
Endovascular Treatment plus 0.125 mg/kg Tenecteplase	0.90 (0.55, 1.47)	0.285	5
Endovascular Treatment plus 100,000 IU Urokinase	0.95 (0.66, 1.37)	0.340	4
**Death within 90 days**			
Endovascular Treatment		0.647	2
Endovascular Treatment plus 0.0313 mg/kg Tenecteplase	0.62 (0.22, 1.53)	0.197	5
Endovascular Treatment plus 0.0625 mg/kg Tenecteplase	0.81 (0.54, 1.21)	0.335	4
Endovascular Treatment plus 0.125 mg/kg Tenecteplase	0.98 (0.54, 1.79)	0.591	3
Endovascular Treatment plus 100,000 IU Urokinase	1.08 (0.69, 1.70)	**0.729**	**1**
**Symptomatic intracranial hemorrhage**			
Endovascular Treatment		0.419	4
Endovascular Treatment plus 0.0313 mg/kg Tenecteplase	1.08 (0.13, 5.88)	0.491	2
Endovascular Treatment plus 0.0625 mg/kg Tenecteplase	1.56 (0.77, 3.21)	**0.788**	**1**
Endovascular Treatment plus 0.125 mg/kg Tenecteplase	0.88 (0.30, 2.58)	0.366	5
Endovascular Treatment plus 100,000 IU Urokinase	0.99 (0.42, 2.38)	0.436	3
**Any intracranial hemorrhage**			
Endovascular Treatment		0.368	4
Endovascular Treatment plus 0.0313 mg/kg Tenecteplase	1.18 (0.53, 2.56)	0.577	2
Endovascular Treatment plus 0.0625 mg/kg Tenecteplase	**1.44 (1.03, 2.02)**	**0.876**	**1**
Endovascular Treatment plus 0.125 mg/kg Tenecteplase	0.84 (0.48, 1.48)	0.207	5
Endovascular Treatment plus 100,000 IU Urokinase	1.06 (0.72, 1.56)	0.471	3

(b) Subgroup analyses about the mixed subgroup (IVT + EVT + IAT/ EVT+ IAT).

	OR (95% CrI)	SUCRA	Rank
**Excellent outcome (mRS 0–1) at 90 days**			
Endovascular Treatment		0.043	3
Endovascular Treatment plus 0.225 mg/kg Alteplase	**1.95 (1.32, 2.90)**	**0.877**	**1**
Endovascular Treatment plus 0.0625 mg/kg Tenecteplase	1.52 (0.83, 2.78)	0.580	2
**Functional independence (mRS 0–2) at 90 days**			
Endovascular Treatment		0.358	2
Endovascular Treatment plus 0.225 mg/kg Alteplase	1.28 (0.87, 1.88)	**0.864**	**1**
Endovascular Treatment plus 0.0625 mg/kg Tenecteplase	0.92 (0.53, 1.60)	0.278	3
**Favorable outcome (mRS 0–3) at 90 days**			
Endovascular Treatment		0.438	2
Endovascular Treatment plus 0.225 mg/kg Alteplase	1.14 (0.76, 1.70)	**0.730**	**1**
Endovascular Treatment plus 0.0625 mg/kg Tenecteplase	0.93 (0.54, 1.59)	0.332	3
**Death within 90 days**			
Endovascular Treatment		0.331	3
Endovascular Treatment plus 0.225 mg/kg Alteplase	1.24 (0.72, 2.17)	**0.715**	**1**
Endovascular Treatment plus 0.0625 mg/kg Tenecteplase	1.05 (0.57, 1.94)	0.454	2
**Symptomatic intracranial hemorrhage**			
Endovascular Treatment		0.426	2
Endovascular Treatment plus 0.225 mg/kg Alteplase	0.65 (0.23, 1.74)	0.116	3
Endovascular Treatment plus 0.0625 mg/kg Tenecteplase	3.02 (0.81, 14.89)	**0.958**	**1**
**Any intracranial hemorrhage**			
Endovascular Treatment		0.117	3
Endovascular Treatment plus 0.225 mg/kg Alteplase	1.18 (0.79, 1.79)	0.448	2
Endovascular Treatment plus 0.0625 mg/kg Tenecteplase	2.00 (0.99, 4.15)	**0.935**	**1**

(c) Subgroup analyses about the OTP ≤6 h subgroup.

	OR (95% CrI)	SUCRA	Rank
**Excellent outcome (mRS 0–1) at 90 days**			
Endovascular Treatment		0.143	4
Endovascular Treatment plus 0.225 mg/kg Alteplase	**2.15 (1.02, 4.64)**	**0.880**	**1**
Endovascular Treatment plus 0.0313 mg/kg Tenecteplase	1.14 (0.53, 2.41)	0.332	3
Endovascular Treatment plus 0.0625 mg/kg Tenecteplase	1.51 (0.94, 2.43)	0.645	2
**Functional independence (mRS 0–2) at 90 days**			
Endovascular Treatment		0.471	3
Endovascular Treatment plus 0.225 mg/kg Alteplase	1.18 (0.54, 2.58)	**0.657**	**1**
Endovascular Treatment plus 0.0313 mg/kg Tenecteplase	0.89 (0.44, 1.82)	0.344	4
Endovascular Treatment plus 0.0625 mg/kg Tenecteplase	1.03 (0.66, 1.61)	0.528	2
**Favorable outcome (mRS 0–3) at 90 days**			
Endovascular Treatment		0.446	4
Endovascular Treatment plus 0.225 mg/kg Alteplase	1.04 (0.45, 2.41)	0.511	2
Endovascular Treatment plus 0.0313 mg/kg Tenecteplase	1.07 (0.50, 2.36)	**0.545**	**1**
Endovascular Treatment plus 0.0625 mg/kg Tenecteplase	1.03 (0.66, 1.62)	0.499	3
**Death within 90 days**			
Endovascular Treatment		**0.731**	**1**
Endovascular Treatment plus 0.225 mg/kg Alteplase	0.48 (0.13, 1.56)	0.193	4
Endovascular Treatment plus 0.0313 mg/kg Tenecteplase	0.67 (0.24, 1.70)	0.360	3
Endovascular Treatment plus 0.0625 mg/kg Tenecteplase	1.00 (0.59, 1.66)	0.717	2
**Symptomatic intracranial hemorrhage**			
Endovascular Treatment		0.457	3
Endovascular Treatment plus 0.225 mg/kg Alteplase	**0.00 (0.00, 0.20)**	0.004	4
Endovascular Treatment plus 0.0313 mg/kg Tenecteplase	1.50 (0.17, 9.61)	0.636	2
Endovascular Treatment plus 0.0625 mg/kg Tenecteplase	2.69 (0.93, 8.93)	**0.903**	**1**
**Any intracranial hemorrhage**			
Endovascular Treatment		0.346	3
Endovascular Treatment plus 0.225 mg/kg Alteplase	0.85 (0.38, 1.89)	0.246	4
Endovascular Treatment plus 0.0313 mg/kg Tenecteplase	1.21 (0.53, 2.72)	0.568	2
Endovascular Treatment plus 0.0625 mg/kg Tenecteplase	1.52 (0.89, 2.61)	**0.840**	**1**

(d) Subgroup analyses about the OTP ＞6 h subgroup.

	OR (95% CrI)	SUCRA	Rank
**Excellent outcome (mRS 0–1) at 90 days**			
Endovascular Treatment		0.085	4
Endovascular Treatment plus 0.0625 mg/kg Tenecteplase	1.22 (0.87, 1.72)	0.488	2
Endovascular Treatment plus 0.125 mg/kg Tenecteplase	**1.91 (1.13, 3.28)**	**0.942**	**1**
Endovascular Treatment plus 100,000 IU Urokinase	1.22 (0.87, 1.73)	0.485	3
**Functional independence (mRS 0–2) at 90 days**			
Endovascular Treatment		0.391	4
Endovascular Treatment plus 0.0625 mg/kg Tenecteplase	1.11 (0.79, 1.57)	**0.655**	**1**
Endovascular Treatment plus 0.125 mg/kg Tenecteplase	0.98 (0.60, 1.59)	0.396	3
Endovascular Treatment plus 100,000 IU Urokinase	1.06 (0.76, 1.49)	0.558	2
**Favorable outcome (mRS 0–3) at 90 days**			
Endovascular Treatment		0.445	2
Endovascular Treatment plus 0.0625 mg/kg Tenecteplase	1.33 (0.92, 1.94)	**0.907**	**1**
Endovascular Treatment plus 0.125 mg/kg Tenecteplase	0.90 (0.55, 1.47)	0.293	4
Endovascular Treatment plus 100,000 IU Urokinase	0.95 (0.66, 1.36)	0.355	3
**Death within 90 days**			
Endovascular Treatment		0.582	2
Endovascular Treatment plus 0.0625 mg/kg Tenecteplase	0.79 (0.51, 1.24)	0.205	4
Endovascular Treatment plus 0.125 mg/kg Tenecteplase	0.98 (0.54, 1.77)	0.527	3
Endovascular Treatment plus 100,000 IU Urokinase	1.08 (0.69, 1.69)	**0.687**	**1**
**Symptomatic intracranial hemorrhage**			
Endovascular Treatment		0.425	3
Endovascular Treatment plus 0.0625 mg/kg Tenecteplase	1.46 (0.68, 3.20)	**0.789**	**1**
Endovascular Treatment plus 0.125 mg/kg Tenecteplase	0.87 (0.29, 2.54)	0.348	4
Endovascular Treatment plus 100,000 IU Urokinase	0.99 (0.42, 2.35)	0.438	2
**Any intracranial hemorrhage**			
Endovascular Treatment		0.372	3
Endovascular Treatment plus 0.0625 mg/kg Tenecteplase	**1.54 (1.07, 2.21)**	**0.954**	**1**
Endovascular Treatment plus 0.125 mg/kg Tenecteplase	0.84 (0.48, 1.47)	0.189	4
Endovascular Treatment plus 100,000 IU Urokinase	1.06 (0.72, 1.57)	0.484	2
